# Endocytosed HIV-1 Envelope Glycoprotein Traffics to Rab14^+^ Late Endosomes and Lysosomes to Regulate Surface Levels in T-Cell Lines

**DOI:** 10.1128/jvi.00767-22

**Published:** 2022-06-30

**Authors:** Huxley K. Hoffman, Rebekah S. Aguilar, Austin R. Clark, Nicholas S. Groves, Nairi Pezeshkian, Merissa M. Bruns, Schuyler B. van Engelenburg

**Affiliations:** a Molecular and Cellular Biophysics Program, Department of Biological Sciences, University of Denver, Denver, Colorado, USA; Emory University

**Keywords:** HIV-1, envelope glycoprotein, Env, T cell, endocytosis, trafficking

## Abstract

Production of infectious HIV-1 particles requires incorporation of the viral envelope glycoprotein (Env) at the plasma membrane (PM) of infected CD4^+^ T cells. Env trafficking to the PM exposes viral epitopes that can be exploited by the host immune system; however, HIV-1 can evade this response by endocytosis of excess Env from the PM. The fate of Env after internalization remains unclear, with evidence suggesting several different vesicular trafficking steps may be involved, including recycling pathways. To date, there have been very few studies documenting the trafficking pathways of native Env in infected T cells. Furthermore, it remains unclear whether there are T-cell-specific endosomal pathways regulating the fate of endocytic Env. Here, we use a pulse-labeling approach with a monovalent anti-Env Fab probe to characterize the trafficking of internalized Env within infected CD4^+^ T-cell lines, together with CRISPR/Cas9-mediated endogenous protein tagging, to assess the role of host cell Rab GTPases in Env trafficking. We show that endocytosed Env traffics to Rab14^+^ compartments that possess hallmarks of late endosomes and lysosomes. We also demonstrate that Env can recycle back to the PM, although we find that recycling does not occur at high rates when compared to the model recycling protein transferrin. These results help to resolve open questions about the fate and relevance of endocytosed Env in HIV-infected cells and suggest a novel role for Rab14 in a cell-type-specific late-endosomal/lysosomal trafficking pathway in T cells.

**IMPORTANCE** HIV-1 envelope glycoprotein (Env) evades immune neutralization through many mechanisms. One immune evasion strategy may result from the internalization of excess surface-exposed Env to prevent antibody-dependent cellular cytotoxicity or neutralization. Characterization of the fate of endocytosed Env is critical to understand which vesicular pathways could be targeted to promote display of Env epitopes to the immune system. In this study, we characterize the endocytic fate of native Env, expressed from infected human T-cell lines. We demonstrate that Env is rapidly trafficked to a late-endosome/lysosome-like compartment and can be recycled to the cell surface for incorporation into virus assembly sites. This study implicates a novel intracellular compartment, marked by host-cell Rab14 GTPases, for the sequestration of Env. Therapeutic approaches aimed at mobilizing this intracellular pool of Env could lead to stronger immune control of HIV-1 infection via antibody-dependent cell-mediated cytotoxicity.

## INTRODUCTION

An important mechanism by which human immunodeficiency virus type 1 (HIV-1) evades the host immune response is by restricting the levels of HIV-1 envelope glycoprotein (Env) on the surface of infected cells and on virus particles (1–4). The Env spikes on HIV-1 particles are required to bind to the CD4 receptor and CCR5 or CXCR4 coreceptor on a target T cell to drive fusion of the viral and host cell membranes (5). Env incorporation into HIV-1 particles during virus assembly, occurring at the plasma membrane (PM) of infected cells, is therefore essential for viral infectivity. However, surface-exposed Env is a target for the host immune response; thus, the surface expression of HIV-1 Env may be regulated to balance the pressures of immune evasion and viral infectivity. Understanding the mechanisms that regulate Env cell surface expression and virus incorporation should inform the development of novel therapeutic approaches targeting these processes.

**FIG 1:**
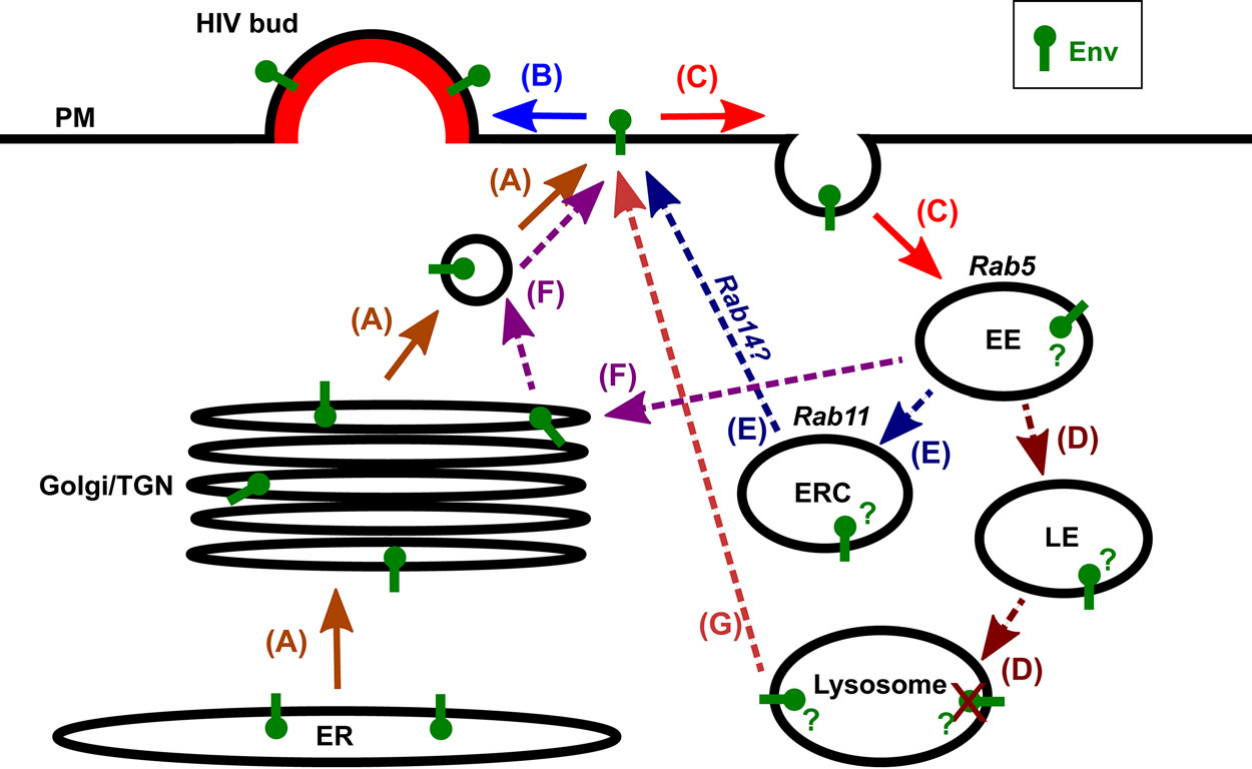
Hypothetical routes of intracellular trafficking for HIV-1 envelope glycoprotein (Env). (A to C) HIV-1 Env biosynthesis, secretion, virus incorporation, and endocytosis. Env is synthesized on the ER and trafficked through the Golgi complex and trans-Golgi network (TGN), following the secretory pathway to the PM (A). (B and C) At the PM, Env can be incorporated in the Gag lattice of an assembling virus particle (B) or internalized by endocytosis (C). Endocytosed cargos are generally delivered to the early endosome (EE), which is regulated by Rab5. (D to G) Several different proposed postendocytic pathways for Env. (D) Trafficking of endocytosed Env via the late endosome (LE) to the lysosome for degradation. (E) Recycling of endocytosed Env via the endosomal recycling compartment (ERC), dependent on Rab14 (31, 32). Rab11 defines and regulates the ERC. (F) Retrograde trafficking of endocytosed Env to the Golgi/TGN (33, 34). (G) Regulated secretion of endocytosed Env via secretory lysosomes (35).

HIV-1 Env is synthesized in the endoplasmic reticulum (ER), processed through the Golgi complex and trans-Golgi network (TGN), and trafficked through the secretory pathway to the PM (reviewed in reference 2) (Fig. 1A). The mature Env spike is a heterodimeric trimer formed between three surface glycoprotein gp120 and three transmembrane gp41 polypeptides. The gp41 subunit has a long C-terminal cytoplasmic tail (CT) that is believed to mediate trafficking and incorporation at HIV-1 assembly sites. Motifs in the gp41 CT mediate Env endocytosis, trafficking, and virus incorporation (6–11). The CT is required for Env incorporation and HIV-1 replication in most physiologically relevant T-cell lines but is dispensable in other cell lines such as HeLa (12, 13). The reason for this cell-type Env-CT dependence has recently been suggested to be caused by increased cellular expression of Env and surface display, resulting in passive incorporation of tailless Env (14). At the PM, Env either becomes incorporated in a viral bud formed by HIV-1 Gag (Fig. 1B) (15–17), or it is internalized by clathrin-mediated endocytosis (Fig. 1C) via conserved endocytosis motifs in the gp41 CT (6, 18–20). This internalization of Env from the PM may be an important mechanism for its concealment from the host immune system, as Env-CT mutations that increase Env levels on the cell surface produce more robust antibody responses (4, 21, 22) and promote the killing of infected cells by antibody-dependent cell-mediated cytotoxicity (3). Mutation of a key endocytosis motif in the Env-CT reduces virus infectivity in T cells, yet this same mutation increases cell-surface Env levels (7). Furthermore, mutation of this endocytosis motif impairs cell-to-cell transfer of infectious virus while also increasing cell-to-cell transfer of apparently non-virus-associated Env, indicating that Env endocytosis is important for cell-to-cell spread of HIV-1 (23). The Env protein of the closely related simian immunodeficiency virus (SIV), like HIV-1 Env, also possesses an endocytosis motif in the Env-CT that mediates internalization of SIV Env from the PM when it is not incorporated into virions (24). When the endocytosis motif in SIV Env-CT is deleted, the virus still replicates in macaques to reach an initial acute peak of infection in lymphoid tissue similar to that of wild-type SIV but then appears to be better controlled by the host immune system. Specifically, perturbation to the SIV Env-CT presents *in vivo* with delayed progression to disease in rhesus macaques, and robust control of the infection mediated by CD8^+^ T cells and/or NK cells in pigtail macaques, showing reduced infection in gut tissue and macrophage cells compared to wild-type SIV (25–28). Thus, for SIV as well as for HIV-1, the endocytosis motif in the Env-CT plays a cell-type-dependent role in the spread of infection; however, the specific mechanisms regulating this immune evasion on the cellular level require further study.

Display of the viral antigen Env on the surface of cells infected with HIV-1 is a necessity for virus assembly on the PM. Env endocytosis may represent one solution for the virus to alleviate excess antigen display beyond what is required for viral incorporation and infectivity. It remains unclear, however, whether this potentially excess Env is trafficked to lysosomes and degraded (Fig. 1D) or recycled back to the plasma membrane (Fig. 1E to G). A population of uncleaved or misfolded Env from the biosynthetic pathway has been shown to be degraded in lysosomes (29, 30), but it is not known whether this is also the case for endocytosed Env. Several different studies have shown evidence for different postendocytic trafficking pathways for Env. For example, recent studies from the Spearman group (8, 31, 32) have suggested that Env trafficking through the endosomal recycling compartment (ERC) (Fig. 1E), dependent on Rab11-family interacting protein 1C (FIP1C) and Rab14, is required for Env incorporation in HIV-1 particles. Interestingly, this trafficking of Env to the ERC was found to be specific to HIV-1 Env-CT and not SIV Env-CT (32), indicating that although endocytosis from the PM is a shared feature of both HIV-1 Env and SIV Env, their postendocytic intracellular trafficking pathways may differ. Additional studies (33, 34) have shown retrograde trafficking of endocytosed HIV-1 Env to the Golgi and TGN (Fig. 1F). Jolly et al. (35) showed evidence for endocytosed Env trafficking by a T-cell-specific regulated secretory pathway, via a specialized secretory lysosome (Fig. 1G), for direct cell-cell spread of HIV-1 at sites of cellular contact termed the virological synapse (VS). Collectively, these studies show that, upon endocytosis, Env has the potential to traverse many endosomal pathways, yet very few molecular players have been identified that may be regulating these trafficking pathways.

Several important methodological limitations have obstructed our view of the Env endocytic pathway. First, probes of endogenous host cell factors regulating the endosomal pathway have not been created to study Env endosomal trafficking in T cells. Second, many studies have been performed in epithelial model cell lines such as HeLa, which may not accurately represent the trafficking pathways active in CD4^+^ T cells, the main targets for HIV-1 infection *in vivo* (8, 31). Finally, due to the difficulty of specifically labeling endocytosed Env, many experiments have relied either on bulk staining for total intracellular Env, in which endocytosed Env is obscured by newly biosynthesized Env; pulse-labeling for an Env chimera in which gp41 CT is fused to a synthetic ectodomain (6, 32, 34), which could perturb Env trafficking by perturbing trimerization; or pulse-labeling full-length Env with a bivalent antibody, which has been shown to alter Env endocytosis by cross-linking Env trimers (36). Herein, we attempt to circumvent these previous limitations by characterizing the fate of endocytic HIV-1 Env using probes for both natively expressed Env and endogenous endosomal trafficking regulators. Confocal fluorescence microscopy in CD4^+^ T cell lines, combined with CRISPR/Cas9-mediated endogenous protein tagging and pulse-labeling of native HIV-1 Env with a monovalent Fab fragment probe, was applied to examine the trafficking of endocytosed Env in a physiologically relevant context not accessible to previous studies. We have characterized the intracellular compartments to which HIV-1 Env traffics after endocytosis and the involvement of several endogenous host cell Rab GTPases in this trafficking, finding endocytosed Env localizes to Rab14^+^ compartments, which both microscopically and biochemically have characteristics of late endosomes (LEs) and lysosomes. We also find that Rab14 activity may play a role. We have also addressed whether endocytosed Env recycles to the PM, showing native Env recycling, but additionally finding that it does not occur constitutively at high rates in our system. Finally, we show that the bulk of endocytosed Env remains sequestered upon virological synapse formation using a coculture between macrophage and T-cell lines. Collectively, these results suggest that the majority of endocytosed Env remains sequestered in intracellular compartments in HIV-1-infected T-cell lines, potentially to alleviate excess antigen display on the PM.

## RESULTS

### Endocytosed Env traffics with transferrin through Rab5^+^ early endosomes and into Rab14^+^ compartments.

To address questions about the postendocytic trafficking of HIV-1 Env, we applied a pulse-chase labeling approach to measure Env endocytic trafficking (37). In this assay, live unpermeabilized HIV-infected cells of the human CD4^+^ T-cell line CEM-A (38) were pulsed with fluorescently labeled anti-gp120 monovalent Fab fragments to stain surface-exposed Env, incubated for a chase time to allow a pool of the stained Env to be internalized and trafficked intracellularly, and then fixed and analyzed by confocal fluorescence microscopy. This pulse-chase approach enables specific detection of surface-exposed and endocytosed Env, in contrast to staining for total Env in permeabilized cells, where the pool of newly biosynthesized Env in the ER and Golgi would obscure detection of endocytosed Env. The use of a monovalent Fab probe rather than a full IgG antibody is important, avoiding cross-linking of Env trimers, because bivalent binding by a full IgG has been shown to induce Env internalization (36).

**FIG 2:**
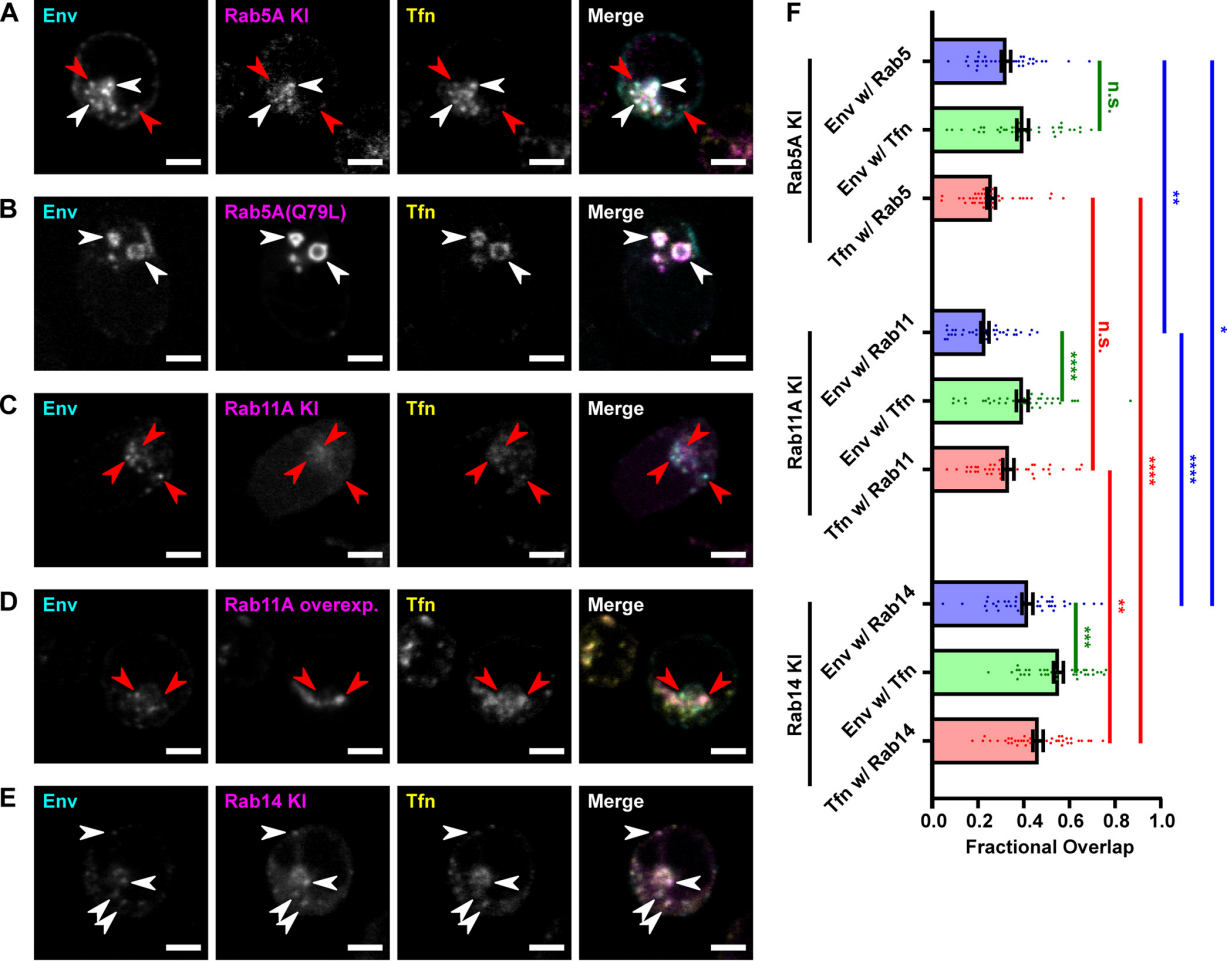
Endocytosed HIV-1 Env traffics through Rab5^+^ early endosomes and into Rab14^+^ compartments. (A to E) HIV-1-infected CEM-A T cells were pulsed with probes for Env and transferrin (Tfn) for 15 min at 4°C, then chased at 37°C, fixed, and imaged by confocal fluorescence microscopy. Representative confocal slices are shown. All scale bars 5 μm. White arrows indicate examples of colocalization, while red arrows indicate examples of noncolocalization. (A) mRuby3-Rab5A KI CEM-A cells were infected, pulse-labeled with anti-Env Fab b12-AF647 and Tfn-AF488, and chased for 1 h. Env and Tfn partially colocalized with Rab5A. (B) Wild-type (WT) CEM-A cells were infected with HIV-1 virus bearing a Rab5A(Q79L) expression cassette in replacement of the *nef* open reading frame, expressing GFP-Rab5A(Q79L) in place of HIV-1 Nef protein. The cells were pulse-labeled with anti-Env Fab b12-Atto565 and Tfn-AF647, and chased for 20 min. Both Env and Tfn accumulated in Rab5A(Q79L)-induced swollen EEs. (C) HIV-1-infected GFP-Rab11A KI CEM-A cells were pulse-labeled with anti-Env Fab b12-AF647 and Tfn-AF555, and chased for 1 h. Env showed little overlap with Rab11, and Tfn overlapped partially with Rab11, but Tfn colocalized more so with Env. (D) CEM-A cells stably overexpressing high levels of TagRFP-Rab11A were treated as in panel C. Tfn accumulated in the swollen ERC induced by Rab11 overexpression, while Env did not. (E) GFP-Rab14 KI CEM-A cells were treated as in (C). Env and Tfn colocalized significantly with Rab14. (F) Fractional overlap between probes in the assays represented in panels A, C, and E; *n* = 40 infected cells per sample. “Env w/Rab5” as example denotes the percentage of Env-positive pixels that were also positive for Rab5. Bars represent means, error bars represent SE, and points represent the values for individual cells. Statistical significance was assessed by Brown-Forsythe and Welch ANOVA tests and Dunnett’s T3 multiple-comparison test. n.s., *P > *0.05, *, *P ≤ *0.05, **, *P ≤ *0.01, ***, *P ≤ *0.001, ****, *P ≤ *0.0001.

Fluorescently labeled transferrin (Tfn), a classic endocytic recycling cargo (39), was pulsed in with the anti-Env Fab as a control to demonstrate that the endocytic machinery in HIV-1-infected CEM-A cells was functional. Endocytosed Env was observed to colocalize with endocytosed Tfn in these assays (Fig. 2A), consistent with previous observations from our laboratory (37). The intracellular trafficking of Tfn has been extensively studied; after binding to its receptor, Tfn is internalized by clathrin-mediated endocytosis and transported to the early endosome (EE), from which it can be recycled back to the PM either by a fast recycling route directly from the EE or by a slow recycling route via the endocytic recycling compartment (ERC) (39). The colocalization of Env with Tfn therefore suggested that the endocytosed Env was localized in the EE and/or ERC and could be destined for recycling to the PM. To test this hypothesis, we characterized the intracellular localization and trafficking pathway of endocytic Env during a single-round infection.

To identify the subcellular compartments where the endocytosed Env and Tfn were localized, we aimed to identify which cellular Rab GTPases were associated with those compartments. Rab GTPases associate with specific intracellular membrane compartments and regulate different intracellular trafficking pathways (39, 40). As example, Rab5 labels the EE and regulates cargo delivery to the EE (41) while Rab11 marks the ERC and regulates the slow recycling pathway (42).

Overexpression of Rab proteins can perturb the trafficking pathways in which they operate. For example, overexpression of Rab5 increases endocytic uptake (41), and overexpression of Rab11 inhibits slow recycling via the ERC (43). To enable detection of endogenous Rab GTPases of interest, without overexpression, we employed CRISPR/Cas9-mediated endogenous gene tagging (44) to express Rab5A, Rab11A, and Rab14 with fluorescent protein (FP) fusion tags from their native genomic loci in CEM-A CD4^+^ T cells (Fig. S1 in supplemental materials). Rab5A and Rab11A were selected because they are well-characterized regulators of the EE and ERC, respectively (39). Rab14 was selected based on prior evidence showing that Rab14 depletion impairs HIV-1 Env incorporation (31), which suggests a role for Rab14 in endocytic Env trafficking. The use of a CD4^+^ T-cell line is important in order to accurately represent the cellular tropism of HIV-1 infection *in vivo*, reasoning that Env trafficking may be altered in Env cytoplasmic tail-dependent versus -independent cell lines.

Pulse-chase assays were then performed on the FP-Rab knock-in (KI) CEM-A lines to assess the localization of endocytosed Env and Tfn with respect to endogenous Rab5A, Rab11A, and Rab14. Endogenously tagged mRuby3-Rab5A marked small punctate vesicles that were concentrated both around the cell periphery (as indicated by the outer limits of Env staining) and in a perinuclear region (as indicated by the inner limits of Env staining, which is excluded from the nucleus) (Fig. 2A). At a chase time of 1 h, pulse-labeled Env and Tfn were partially colocalized with Rab5A; some Env and Tfn-containing vesicles were Rab5A^+^, identifying them as EEs, but there were also many cargo containing vesicles that did not colocalize with Rab5A. We hypothesized that this was because the majority of endocytosed Env and Tfn had already left the EE by 1 h. To test this hypothesis, the pulse-chase assay for Env and Tfn was performed on CEM-A cells transiently overexpressing green fluorescent protein (GFP)-tagged Rab5A(Q79L), a constitutively active mutant that has been shown to increase endocytic uptake to the EE and inhibit trafficking out of the EE, producing characteristic enlarged EEs (45). GFP-Rab5A(Q79L) overexpression gave the expected phenotype of swollen EEs, accumulating Tfn. We observed that endocytosed Env also accumulated in the Rab5A(Q79L)^+^ EEs (Fig. 2B). These results indicated that endocytosed Env, like Tfn, is transported through Rab5^+^ EEs.

Next, we sought to characterize the compartments downstream of the EE, namely, the ERC, regulated by Rab11. Endogenous GFP-Rab11A from CEM-A KI cells localized predominantly to a perinuclear compartment (Fig. 2C). Env showed little colocalization with Rab11A. Since the presence of Rab11 is a defining characteristic of the ERC, this indicated that the endocytosed Env was not trafficking to the ERC in infected CEM-A T cells under these conditions. The majority of the Tfn signal colocalized with Env, as previously observed, and not with Rab11A, but we did observe a fraction of Tfn colocalized with Rab11A (Fig. 2C), consistent with the well-studied slow recycling pathway of Tfn via the ERC. The GFP fluorescence of endogenously tagged Rab11A was often close to the detection limit on the microscope, so we considered that this experiment might underrepresent the colocalization of Env with endogenous Rab11A. To further test for Env trafficking to the ERC, we also performed the pulse-chase experiment on CEM-A cells stably overexpressing GFP-tagged Rab11A (Fig. 2D). GFP-Rab11A overexpression produced aberrantly large tubular ERCs, and Tfn accumulated in these Rab11A^+^ compartments, but Env did not colocalize with the overexpressed Rab11A. These results suggest that, after leaving the EE, endocytosed Env takes a Rab11A-independent pathway, and the compartment to which it is transported is not the ERC. A portion of Tfn follows this Rab11A-independent pathway with Env, while a portion of Tfn undergoes Rab11A-mediated recycling via the ERC in infected CEM-A T cells.

Next, we tested whether Rab14, another GTPase implicated in receptor recycling, is involved in the Rab11A-independent trafficking pathway utilized by Env. Tagged endogenous GFP-Rab14 labeled peripheral vesicles as well as a perinuclear compartment in CEM-A T cells (Fig. 2E). Env and Tfn colocalized strongly with GFP-Rab14, both in vesicles and in the perinuclear compartment. Quantification of the fractional overlap between Env, Tfn, and each of the endogenously tagged Rabs in these assays (Fig. 2F) confirmed that the colocalization of Env with Rab14 was significantly greater than with Rab5, which in turn was significantly greater than with Rab11. Tfn also colocalized significantly more with tagged Rab14 than with Rab5 or Rab11, and Env colocalized as much or significantly more with Tfn as with the Rab in each sample. These findings suggest a role for Rab14 in the trafficking of endocytosed Env in HIV-1-infected CEM-A T cells and are consistent with previous findings (31).

To confirm that the internalized Env detected in Rab14+ organelles represents functional Env trimers, we repeated the pulse-chase labeling of Env in HIV-1-infected CEM-A cells, this time using a quaternary-specific anti-Env Fab, PGT151 (46), which binds only to mature cleaved Env trimers, and imaged the live cells by fluorescence confocal microscopy. Env pulse-labeled with PGT151 Fab colocalized with endogenously expressed GFP-Rab14 (Fig. S2A). This result disfavors the possibility that the Env observed to colocalize with Rab14 in pulse-chase experiments could be a nonfunctional (e.g., uncleaved or nontrimeric) form of Env.

Rab14 is associated with a variety of different intracellular trafficking pathways and compartments. As example, Rab14 has been shown to localize to EEs, the Golgi complex, and TGN and functions in trafficking between endosomes and the Golgi/TGN in epithelial-like cells (47). Rab14 has also been identified as defining an intermediate endosomal recycling compartment, downstream of the Rab5^+^ EE and upstream of the Rab11^+^ ERC (48), and localizes to GLUT4 storage vesicles, playing a role in regulated secretion of insulin (49). The observation that endocytosed Env trafficked to Rab14^+^ compartments suggests that additional steps for routing endocytic Env could be occurring in T cells. To better characterize Rab14^+^ Env compartments, we proceeded to assess the localization of pulse-labeled endocytosed Env and Tfn with respect to probes for intracellular organelles associated with the secretory and endocytic system.

### Endocytosed Env traffics to late endosomes and lysosomes.

**FIG 3:**
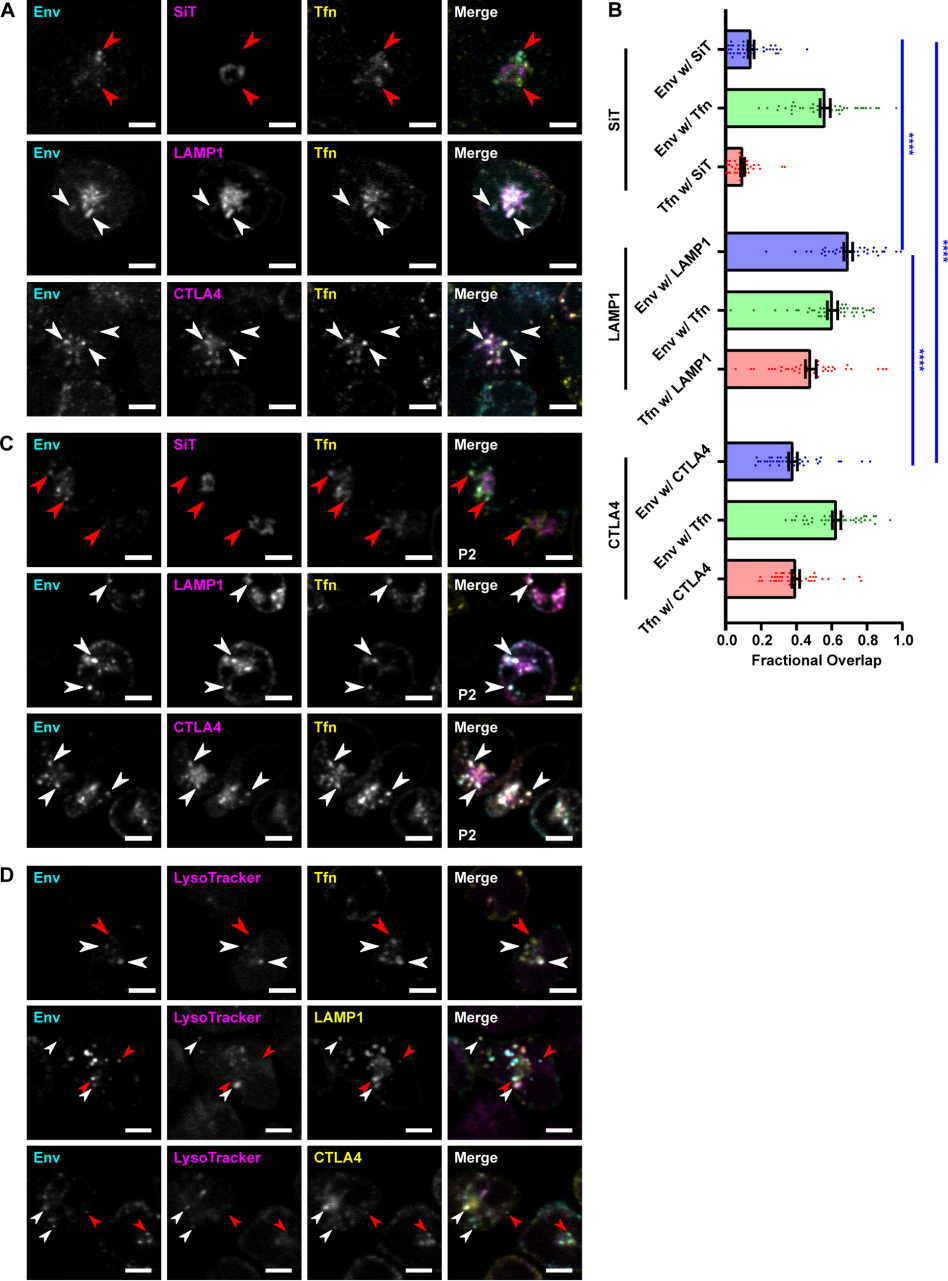
Endocytosed HIV-1 Env traffics to LEs and lysosomes in T cells. (A) WT CEM-A T cells were infected with HIV-1 viruses possessing either a sialyltransferase 1-green fluorescent protein (SiT-GFP), lysosomal associated membrane protein 1 (LAMP1)-Emerald, or CTLA4-GFP expression cassette in replacement of the *nef* gene, which respectively express an fluorescent protein (FP)-tagged Golgi/TGN marker (SiT), LE/lysosome marker (LAMP1), or secretory lysosome cargo (CLTA4) in place of HIV-1 Nef protein. The cells were pulsed with anti-Env Fab b12-AF647 and Tfn-AF555 for 15 min at 4°C, then chased at 37°C for 1 h, fixed, and imaged by confocal fluorescence microscopy. Endocytosed Env and Tfn colocalized significantly with LAMP1, partially with CTLA4, and very slightly with SiT. (B) Fractional overlap between probes in the assays represented in panel A. “Env w/SiT” as example denotes the percentage of Env-positive pixels that were also positive for SiT. Bars represent means, error bars represent SE, and points represent the values for individual cells. Statistical significance was assessed by Brown-Forsythe and Welch ANOVA tests and Dunnett's T3 multiple-comparison test. ****, *P ≤ *0.0001. (C) Assays as in panel A were performed on P2 T cells. Similarly, endocytosed Env and Tfn colocalized well with LAMP1, partially with CTLA4, and to a far lesser extent with SiT. (D) WT CEM-A T cells were infected with WT HIV-1 virus (top) or LAMP1-Emerald (middle) and CTLA4-GFP (bottom) reporter viruses. The cells were pulsed with anti-Env Fab b12-AF647 and Tfn-AF488 (top) or with anti-Env Fab b12-AF647 alone (middle and bottom) for 15 min at 4°C and then chased at 37°C for 1 h with LysoTracker Red DND-99 pulsed for the final 50 min. The cells were fixed and imaged by confocal fluorescence microscopy. LysoTracker labeled a subset of the compartments that were positive for LAMP1 or CTLA4. Scale bars are 5 μm. White arrows indicate examples of colocalization, while red arrows indicate examples of noncolocalization.

First, we tested whether the Rab14^+^ Env-containing compartments represented the Golgi apparatus or TGN. The Golgi localization domain of Golgi/TGN resident enzyme beta-galactoside alpha-2,6-sialyltransferase 1 (SiT) was fused to GFP and transiently expressed in HIV-1-infected CEM-A T cells. The SiT probe localized strictly to a perinuclear compartment, which typically displayed a ring-like morphology in CEM-A cells (Fig. 3A, top). Pulse-labeled Env and Tfn showed a distinct lack of colocalization with the SiT probe. This indicated that the compartments in which internalized Env was detected were not the Golgi or TGN; however, endocytic Env may retrograde traffic through the Golgi or TGN rapidly and not result in a steady-state accumulation in these secretory compartments.

Next, we tested whether the Env-containing compartments might be LEs or lysosomes. FP-tagged lysosomal associated membrane protein 1 (LAMP1), which localizes to LEs and lysosomes (50), was transiently overexpressed as a probe for these compartments. LAMP1 labeled perinuclear compartments and peripheral vesicles in infected CEM-A T cells (Fig. 3A, middle). Internalized Env and Tfn colocalized strongly with LAMP1, indicating that populations of both Env and Tfn trafficked to LEs and lysosome-like compartments after endocytosis. Pulse-chase staining with the quaternary-specific anti-Env Fab PGT151 confirmed the trafficking of mature Env trimers to LAMP1^+^ compartments in CEM-A cells (Fig. S2B), and this was also similar in the suspension T-cell line SupT1 (51) (Fig. S2C), indicating that the above findings are consistent in different T-cell lines.

Cargo transport from the EE to the LE and then to the lysosome is typically a default pathway for degradation (39). However, in addition to their degradative function, lysosomes also function as regulated secretory granules in certain immune cell types (52), including CD4^+^ T cells (53), and there is evidence for HIV-1 Env trafficking via this secretory lysosome pathway (35). To explore the possibility that Env-containing vesicles were secretory lysosomes, we also looked for colocalization with cytotoxic T lymphocyte antigen-4 (CTLA4), which is a cargo of secretory lysosomes in CD4^+^ T cells (53). Transiently overexpressed FP-tagged CTLA4 displayed a distribution similar to that of LAMP1 compartments, with internalized Env and Tfn also colocalizing substantially with CTLA4 vesicles (Fig. 3A, bottom). As CTLA4 is delivered to the cell surface by secretion of CTLA4-containing lysosomes upon immune stimulation (53), the colocalization of endocytosed Env with CTLA4 supports the hypothesis that Env could return to the PM via the secretory lysosome mechanism.

Quantification of fractional overlap confirmed that endosomal Env in CEM-A T cells colocalizes strongly with LAMP1, partially with CTLA4, and very little with SiT under our pulse-chase conditions (Fig. 3B). We additionally tested whether the transport of endocytosed Env to LEs and lysosomes was consistent across CD4^+^ T-cell lines by repeating these experiments in P2 cells, a suspension T-cell line, which has a polarized morphology like that of T cells *in vivo* (54). In P2 cells, as in CEM-A cells, internalized Env and Tfn largely colocalized with LAMP1 and CTLA4, and not with SiT, indicating localization in LEs/lysosomes and little steady-state accumulation in the Golgi/TGN (Fig. 3C).

To further characterize the composition of LAMP1^+^ Env-containing compartments, we used an acidotropic dye, LysoTracker Red DND-99, which accumulates in low-pH organelles representing LEs and lysosomes. LysoTracker labeled a few small vesicles per cell. Internalized Env and Tfn often colocalized with LysoTracker, confirming their transport to LEs or lysosomes, although there were also vesicles of Env and Tfn that did not display LysoTracker labeling (Fig. 3D, top). The vesicles labeled by LysoTracker were a subset of those labeled by LAMP1 (Fig. 3D, middle), and colocalization of endocytosed Env with LAMP1 was observed both on LysoTracker-positive and -negative compartments. Similarly, a subset of CTLA4-containing vesicles were acidic organelles as indicated by LysoTracker staining (Fig. 3D, bottom), and Env colocalization with CTLA4 was detected both in LysoTracker-positive and -negative vesicles. This observation is consistent with a population of Env cotrafficking together with CTLA4 along an endocytic pathway to a late endosomal/lysosomal compartment.

Our findings demonstrate that endocytosed Env and Tfn localize in Rab14^+^ compartments and in LEs and lysosomes. Together, these observations suggest that Rab14 might function in late endosomal or lysosomal trafficking in these cells. We therefore sought to investigate the relationship between Rab14 and LE/lysosome markers.

**FIG 4:**
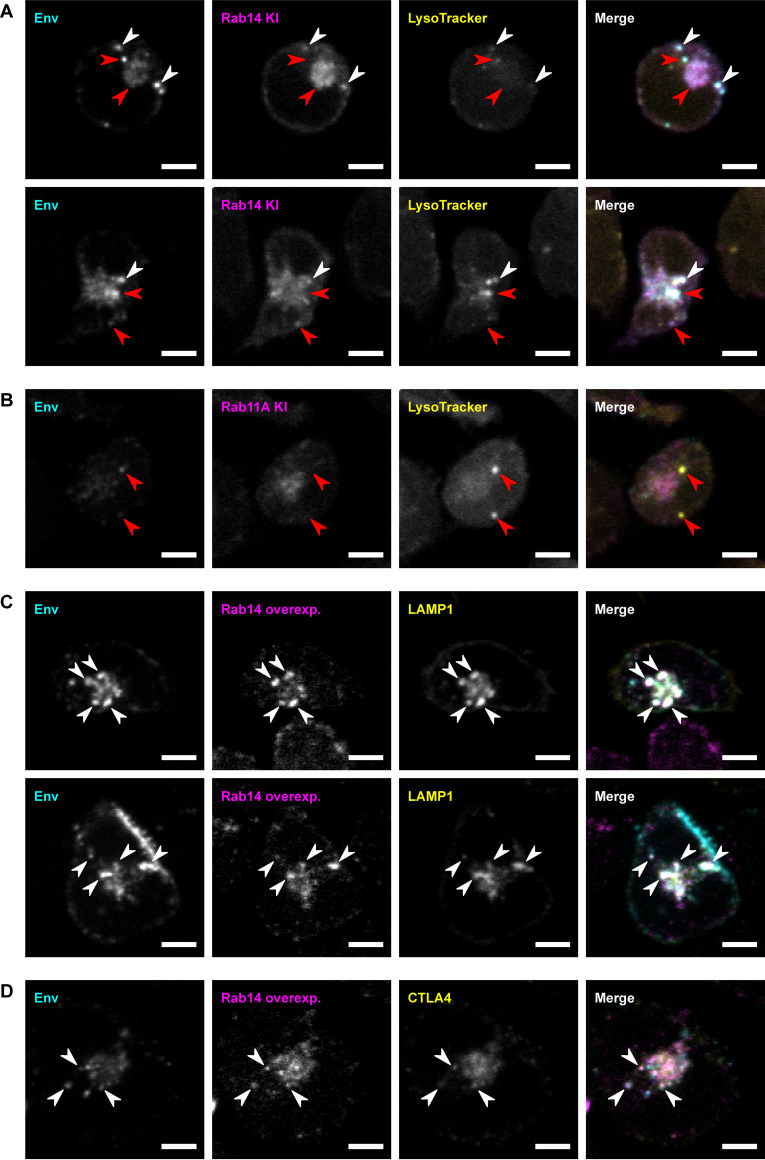
Rab14 resides on late endosomes and lysosomes containing endocytic Env in CEM-A T cells. (A and B) GFP-Rab14 knock-in (KI) or GFP-Rab11A KI CEM-A T cells were infected with HIV-1 virus and pulsed with anti-Env Fab b12-AF647 for 15 min at 4°C and then chased at 37°C for 1 h, with LysoTracker Red DND-99 added in the final 50 min. The cells were fixed and imaged by confocal fluorescence microscopy. Rab14 partially overlapped with LysoTracker, while Rab11 did not. (C and D) CEM-A cells stably overexpressing low levels of TagRFP-Rab14 were infected with HIV-1 virus possessing either LAMP1-Emerald or CTLA4-GFP reporters. The cells were pulsed with anti-Env Fab b12-AF647 for 15 min at 4°C, then chased at 37°C for 1 h, fixed, and imaged by confocal fluorescence microscopy. Rab14 colocalized with LAMP1 and with CTLA4, which also possessed Env staining in many instances. White arrows indicate examples of colocalization, while red arrows indicate examples of noncolocalization. Scale bars are 5 μm.

Partial colocalization was observed between endogenous Rab14 and LysoTracker (Fig. 4A and Fig. S3D). Populations of internalized Env were observed to colocalize with both Rab14 and LysoTracker on the same vesicle (Fig. 4A), while other Env-containing vesicles displayed LysoTracker signal but lacked Rab14 labeling, or the converse. These observations suggest that Rab14^+^ vesicles transport Env to or from LEs or lysosomes. In contrast, endogenously tagged Rab11A did not colocalize with LysoTracker, and internalized Env was more colocalized with LysoTracker than with Rab11A (Fig. 4B), supporting the trafficking of Env to lysosome-like compartments and not to the ERC in T-cell lines.

In CEM-A cells stably overexpressing FP-tagged Rab14 (Fig. 4C), as well as in CEM-A cells with endogenously tagged Rab14 (Fig. S3A and D), or P2 cells overexpressing tagged Rab14 (Fig. S3B), Rab14 and LAMP1 appeared frequently on the same compartments, indicating that LEs and lysosomes are a primary site for Rab14 function in these cells. The compartments in which internalized Env was detected, both perinuclear compartments and peripheral vesicles, were mostly positive for both Rab14 and LAMP1, further supporting a role for Rab14 in the late endosomal/lysosomal trafficking of Env. Overexpressed Rab14 also colocalized substantially with CTLA4 (Fig. 4D), and many, though not all, vesicles containing internalized Env were positive for both Rab14 and CTLA4, consistent with a potential role for Rab14 in trafficking via the secretory lysosome pathway. Collectively, these results support a role for Rab14 in the regulation of transport of endocytosed Env to and/or from LEs and lysosomes in CD4^+^ T-cell lines.

To further assess the role of Rab14 in Env trafficking, we examined the effects of a constitutively active Rab14 mutant, Rab14(Q70L) (47). Rab14(Q70L) showed greater colocalization with LysoTracker^+^ LEs or lysosomes than endogenous Rab14 did, and the colocalization of internalized Env with Rab14(Q70L) was greater than with endogenous Rab14 (Fig. S3C and D). Both wild-type Rab14 and Rab14(Q70L) also showed colocalization with Env in Rab5^+^ EEs (Fig. S3E and F), suggesting that Rab14 is present along with internalized Env throughout the continuum of endosomal compartments from EEs to LEs to lysosomes. Relatively little surface Env signal was observed in pulse-chase labeling of infected CEM-A cells expressing Rab14(Q70L) (Fig. S3C and F; compare to Fig. 4A and 2A). Surface staining for Env on fixed, nonpermeabilized and infected cells confirmed that surface Env levels were significantly decreased by Rab14(Q70L) expression (Fig. S3G and H). Thus, expression of constitutively active Rab14 appears to reduce Env presentation at the cell surface, which could suggest that Rab14 either negatively regulates Env trafficking to the PM or positively regulates endosomal trafficking of Env to terminal compartments unable to recycle to the PM.

**FIG 5:**
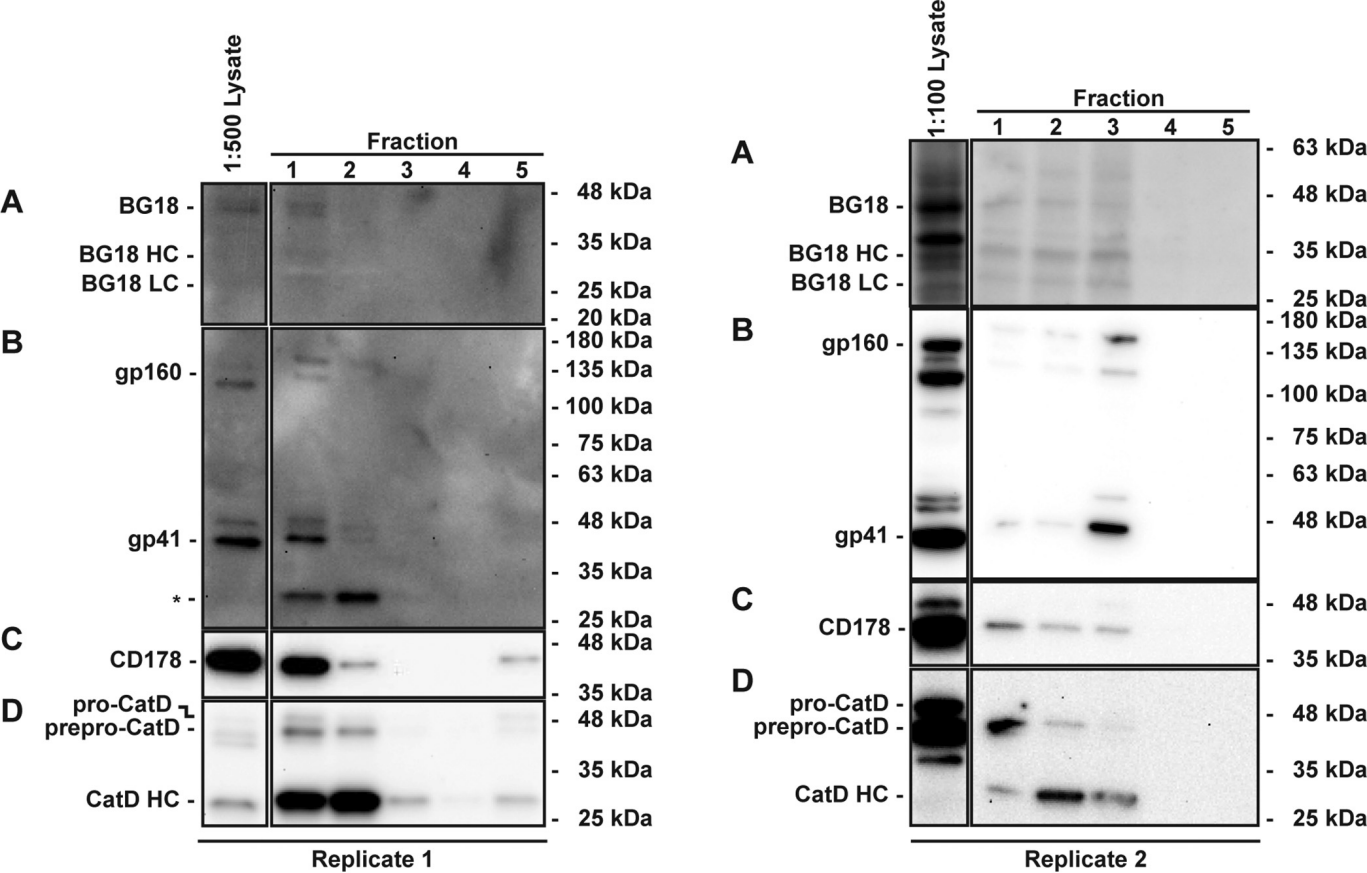
Pulse-chase labeled endocytic Env is biochemically identified within enriched lysosomal fractions isolated from HIV-1-infected CEM-A T cells. Representative Western blots of fractions taken from lysosome isolation and enrichment of HIV-1 infected CEM-A cells. Samples were pulse-chase labeled with anti-Env Fab BG18 and chased for 1.25 h. Lysosomes are enriched within the first two or three fractions in Replicates 1 (left) and 2 (right), respectively. Both anti-Env Fab BG18 bound to Env during pulse-chase, detected with anti-human λ light chain secondary antibody (A) and Env, detected with anti-gp41 Env antibody Chessie8 (B) were found to be enriched in the same fractions as the secretory lysosome cargo CD178/Fas ligand (C) and (D) the lysosomal acid hydrolase Cathepsin D (CatD) (D); HC, heavy chain; LC, light chain). Replicate 1 displays an unidentified lower molecular weight form of gp41 that could represent proteolysis or posttranslational modification (B, asterisk), however, this band was not observed in all replicates. Total lysate versus lysosome enrichment fractions for Cathepsin D show a majority of processed HC hydrolase suggesting these fractions contain active lysosomal components. See Fig. S4 for full blot images and detailed descriptions.

As an orthogonal experimental approach to test for trafficking of endocytosed Env to lysosomes, we performed a biochemical analysis of enriched lysosomal fractions isolated from HIV-infected CEM-A cells in which Env was pulse-labeled similarly as in the pulse-chase assay described above. Whereas the pulse-chase experiments shown above used the anti-Env Fab b12 (55), this experiment used a different anti-Env Fab, BG18 (56, 57), so as to confirm that this finding was also independent of the specific probe chosen to label Env. The BG18 Fab probe along with Env gp41 was detected in fractions that were also enriched for the lysosomal protease Cathepsin D (58) and the secretory lysosome cargo CD178/FasL (59) (Fig. 5 and Fig. S4). This biochemical result corroborates the identification of the endocytosed-Env-containing compartments as lysosomes and potentially secretory lysosomes.

### Endocytosed Env populations are retained in cells and recycled to the plasma membrane.

**FIG 6:**
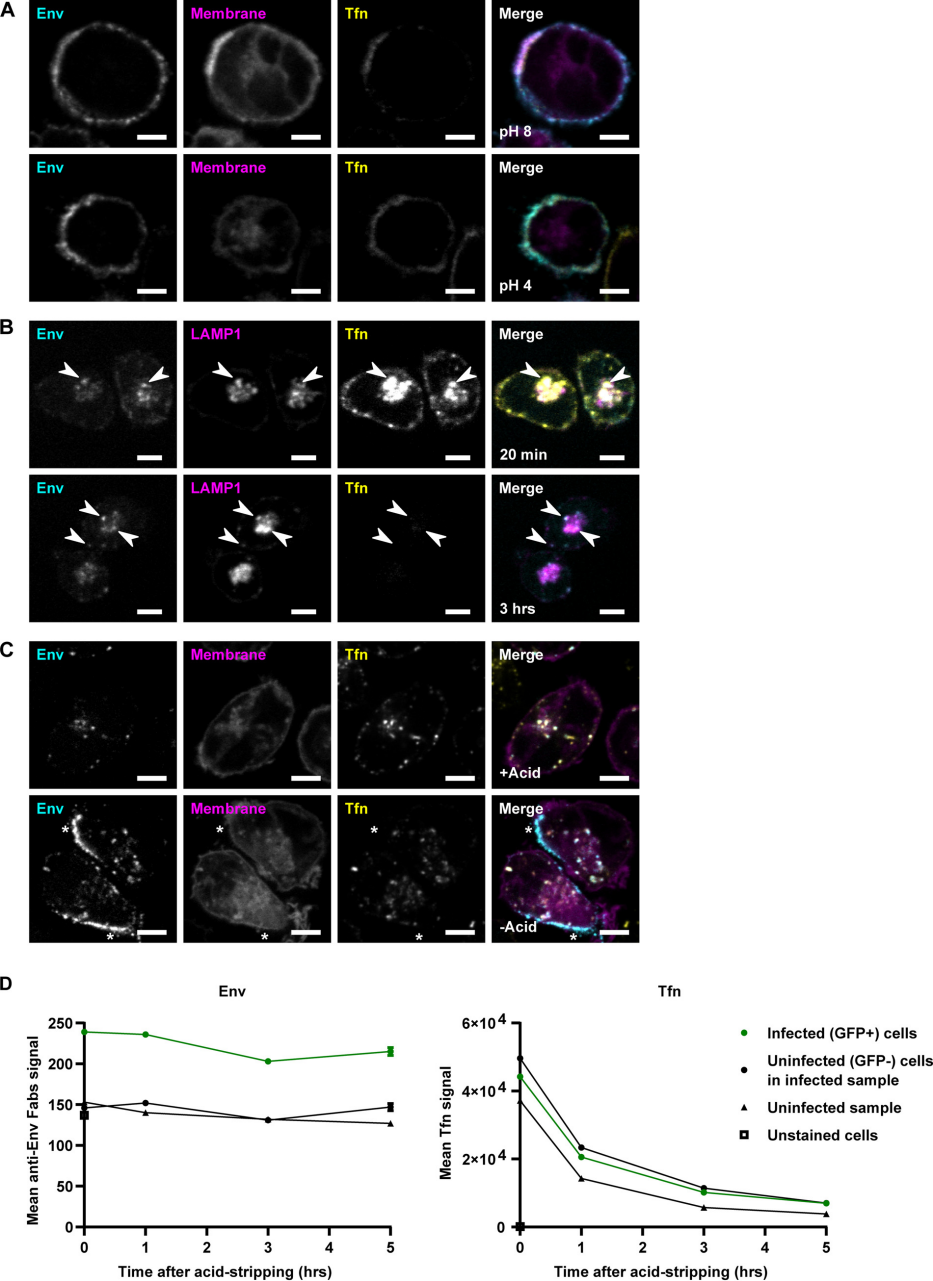
Endocytosed HIV-1 Env is retained in infected cells. (A) WT CEM-A T cells were infected with HIV-1 virus containing a membrane labeling reporter (Membrane, magenta). The cells were pulsed with anti-Env Fab b12-Atto565 and Tfn-AF647 for 15 min at 4°C, then washed either with RPMI media at its standard pH of 8.0, or with RPMI media adjusted to pH 4.0 representing a lower bound for lysosomal pH, for 3 × 5 min at 4°C. Cells were fixed and imaged by confocal fluorescence microscopy. Surface staining was not removed by washing at pH 4.0, indicating lysosomal pH does not dissociate the anti-Env Fab b12 from Env, or Tfn from TfR. (B) WT CEM-A cells were infected with HIV-1 virus possessing a LAMP1-Emerald reporter. The cells were pulsed with anti-Env Fab b12-Atto565 and Tfn-AF647 for 15 min at 4°C, then chased for either 20 min or 3 h at 37°C prior to fixation and imaging by confocal fluorescence microscopy. Internalized Tfn signal decreased dramatically over time, while internalized Env signal did not show such a decrease. (C) WT CEM-A cells were infected with HIV-1 virus possessing a GFP membrane marker (magenta). Cells were pulsed with anti-Env Fab b12-AF647 and Tfn-AF555 for 12 min at 37°C. The samples (top) were treated with an acid solution of 0.2 M acetic acid, 0.5 M NaCl, pH 3, for 1 min at 4°C to dissociate (strip) the surface exposed b12 probes from Env, while other samples (bottom) were kept at physiological pH. The cells were then fixed immediately, without chase time, and imaged by confocal fluorescence microscopy. *Examples of surface Env stain in the non-acid-stripped sample, which was absent in the acid-stripped sample, showing that the stripping procedure effectively dissociates anti-Env Fab probes from surface-exposed Env. Scale bars are 5 μm. (D) P2 T cells were infected with HIV-1 virus harboring a membrane reporter probe to identify infected cells. The cells were simultaneously pulsed with anti-Env Fabs b12-Atto565, BG18-Atto565, and PGT145-Atto565, and Tfn-AF647, for 15 min at 37°C, then acid-stripped as described above for 1 min at 4°C. Samples were then chased for the indicated time periods at 37°C prior to fixation and analyzed by flow cytometry. Internalized Tfn was mostly lost from the cells over 5 h, while internalized Env showed little decrease over the same time. A small, but detectable amount of nonspecific Fab probe uptake was observed in uninfected controls, however, specific staining was readily resolved by flow-cytometry detection. Error bars representing SE are too small to visualize on the graph (*n* ≥ 17,712 events for all conditions at 0 h; *n* ≥ 3,072 events for all conditions at 1 h; *n* ≥ 1,499 events for all conditions at 3 h; *n* ≥ 418 events for all conditions at 5 h; each event represents an individual cell, all from the same experiment).

Our results indicate that Env is transported to lysosomes and raises the question of whether the anti-Env Fab probes could accurately report on Env trafficking after its arrival at the lysosome, since lysosomes are acidic organelles, and low pH can disrupt antibody-antigen interactions. We therefore tested whether exposure to lysosomal pH would cause the anti-Env Fab b12 to dissociate from Env. To yield high levels of surface-exposed Env for this experiment, CEM-A cells were infected with HIV-1 virus lacking the Env gp41 cytoplasmic tail, a mutation that increases Env cell surface expression (14, 37). The cells were pulse-labeled with b12 Fab and Tfn probes and then washed with growth medium, either at the standard pH of 8.0 (RPMI 1640) or adjusted to pH 4.0, representing an approximate lower bound of reported values for lysosomal pH (~4.5 to 5.0) (60–62). Exposure to pH 4.0 did not remove b12 Fab or Tfn staining from the cell surface (Fig. 6A). This result validates the use of Fab-based fluorescent probes for pulse-chase labeling of Env and suggests this probe remains bound to the ectodomain of Env even after reaching the acidic lumen of the lysosome.

Next, we wished to investigate the fate of internalized Env populations over time. The pulse-chase assays thus far demonstrated LE/lysosomal localization of endocytosed Env after 1 h of endocytic trafficking. We compared the results at a shorter chase time of 20 min and at a longer chase time of 3 h after pulse-labeling with b12 Fab and Tfn probes (Fig. 6B). Previous studies have established that Tfn is released back into the medium from its receptor (TfR) upon recycling of the receptor-ligand complex to the cell surface (63–65). Tfn staining was therefore expected to be lost from intracellular compartments over time, and indeed, we observed a dramatic decrease in the intensity of Tfn signal over 3 h. Antibody-based probes such as the anti-Env Fab b12, on the other hand, should not dissociate from their antigen-targets upon return to the cell surface, but lysosomal degradation of the target protein is expected to result in loss of the Fab probe signal from the cells. The decline of signal over time after pulse-labeling with an antibody has been used to measure the lysosomal degradation of CTLA4 (66). The intensity of signal for endocytosed Env, while highly variable between infected cells in biological replicates, did not display a clear decrease in signal intensity over 3 h. Colocalization of endocytosed Env with LAMP1 was apparent at each of these time points. These observations indicate that internalized Env is retained in LEs or lysosomes and that the bulk of endocytic Env is not degraded within 3 h.

To facilitate quantitative measurements of changes in endocytosed Env over time, we modified our pulse-chase assay to include an acid-stripping step after staining to remove the anti-Env Fab associated with immobilized surface exposed Env remaining at the cell surface after the chase time point. This procedure ensures that Fab signal associated with cells represents only endocytosed Env populations. Washing with an acidic solution of pH ~3 for 1 min at 4°C was found to effectively strip the anti-Env Fab probe from the cell surface, leaving only intracellular Env stained (Fig. 6C). Using flow cytometry, we measured levels of pulse-labeled Env and Tfn over time. HIV-1-infected P2 T cells were pulse-labeled with anti-Env Fabs and Tfn probe, acid-stripped, chased for varying amounts of time at 37°C and then fixed and analyzed by flow cytometry (Fig. 6D). Consistent with the observations from confocal microscopy, Tfn staining was almost entirely lost over the course of 5 h, while Env staining showed little decrease over the same time. These results showed that, although internalized Env was transported to LEs and lysosomes, which commonly represents a degradative pathway, Fab probe associated with Env was mostly not degraded by the lysosomal-like compartment within 5 h of internalization.

**FIG 7:**
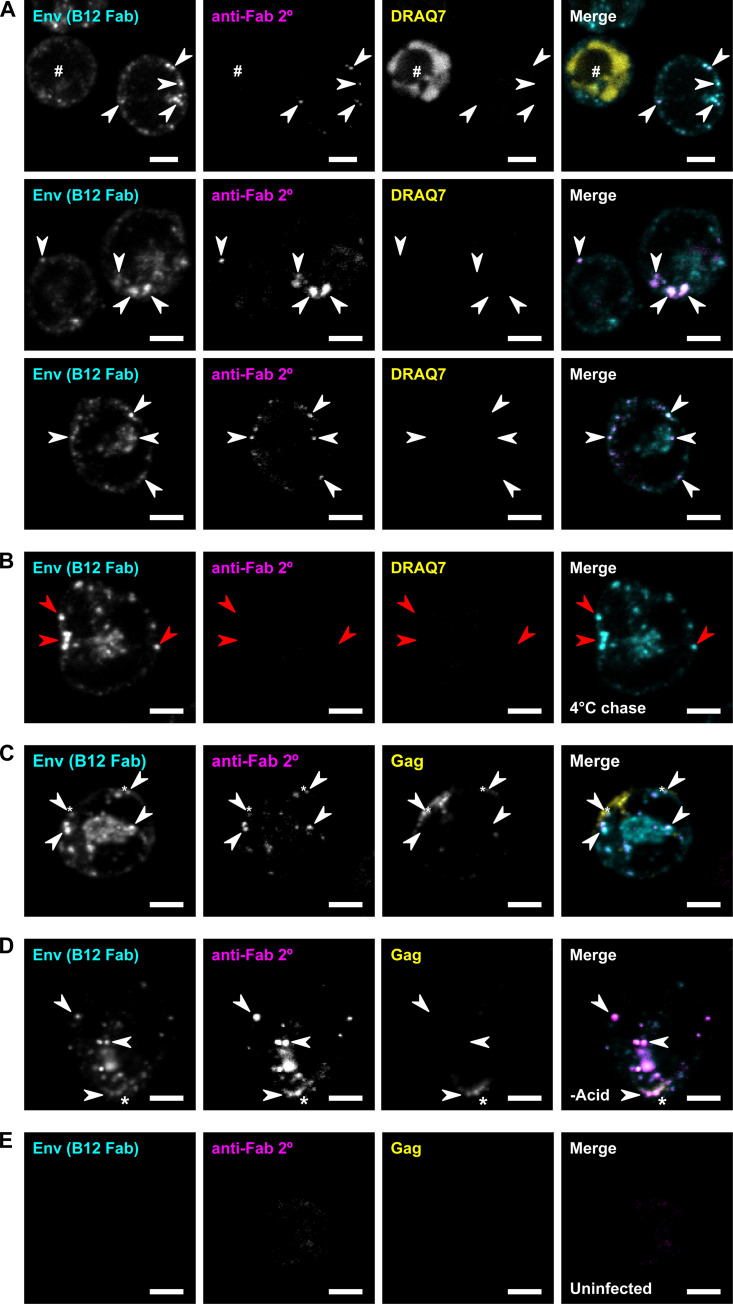
Detection of native endocytic HIV-1 Env recycling to the plasma membrane of infected T cells. (A) WT CEM-A cells were infected with HIV-1 virus and pulse-labeled with anti-Env Fab b12-Atto565 for 20 min at 37°C, and acid-stripped with 0.2 M acetic acid, 0.5 M NaCl, pH 3, for 1 min at 4°C to remove surface-exposed b12 Fab. After 1 h of chase time at 37°C, surface displayed b12 probe that had recycled to the PM was then detected with anti-human kappa light chain secondary antibody labeled with AF488. Cell-impermeant DRAQ7 DNA dye was included during the 1 h chase time to ensure that secondary labeling of human fabs bound to Env was not due to permeabilization of the plasma membrane. The cells were fixed and imaged by confocal fluorescence microscopy. Secondary staining (magenta; white arrows) labels b12 Fab-bound Env that recycled to the PM during the chase time, and DRAQ7 labels the nuclei of any cells are necrotic and the membrane is permeable (#). A fraction of b12-stained Env showed costaining with the secondary anti-human Fab antibody, indicating that this pool of Env-b12 conjugates had recycled after endocytosis. This secondary staining was detected in cells where DRAQ7 was excluded, demonstrating that cell membranes were intact. (B) Cells were treated as in panel A except that the secondary staining was performed at 4°C to prevent recycling. Secondary staining was not detected (red arrows), confirming that the acid-stripping protocol dissociates surface-exposed Fab from Env. (C) WT CEM-A cells were infected with HIV-1 virus harboring a reporter of HIV-1 Gag, which expresses a nanobody probe in the cytoplasm that binds to p24 Gag (CANTD-TagRFP). Cells were pulsed with anti-Env Fab b12-AF647 for 20 min at 37°C, acid-stripped as above for 1 min at 4°C, probed with AF488 anti-human kappa light chain secondary antibody for 1 h at 37°C, fixed, and imaged by confocal fluorescence microscopy. Detectable secondary staining, representing recycled Env, was found to colocalize with HIV Gag at the PM (*). (D) Cells were treated as in panel C except that acid-stripping was omitted, so that secondary staining represents both recycled Env and Env that remained on the PM. (E) Uninfected cells were treated as in panel C, and displayed a lack of both b12 Fab and secondary stain, confirming the specificity of the staining. Maximum intensity projections of five z-slices at 0.3-μm spacing are shown. Scale bars are 5 μm.

We next questioned whether the endocytosed Env remained sequestered inside the cell or whether it ever returned to the cell surface, which would be a prerequisite for it to be incorporated in HIV-1 particles. To test this these possibilities, we further modified the pulse-chase assay with a secondary antibody staining step to detect the recycling of pulse-labeled endocytic Env to the PM (Fig. 7). In this assay, the cells were again pulse-labeled with anti-Env Fab b12 and acid-stripped so that only endocytosed Env was stained. The cells were then chased for 1 h at 37°C in the presence of an AlexaFluor488 dye-labeled anti-human light chain secondary antibody recognizing the human b12 Fab probe. Because the cells were not permeabilized, the secondary antibody can only label-infected cells if the b12-Fab Env complexes recycle back to the cell surface from an internalized endocytic pool. Any secondary staining in this assay would therefore represent Env that had been surface-exposed during the b12 Fab pulse, was internalized prior to acid-stripping, and then returned to the PM during the 1-h chase.

In some of the cells, a fraction of the b12-labeled Env was colabeled with the secondary antibody (Fig. 7A), indicating that this Env had recycled. Secondary staining was detected in cells from which a cell-impermeant (vital) dye was excluded, demonstrating that cell membranes were intact, leaving only the possibility of b12 Fab-Env complexes being recycled to the PM. A control sample, kept at 4°C to halt intracellular trafficking during the chase time (Fig. 7B), did not show secondary staining, indicating that the acid-stripping had effectively removed surface-exposed b12 Fab, thus confirming that secondary staining in the samples incubated at 37°C represented recycled Env. These results, using our Fab-labeled native Env, are consistent with a recent report from the Chen group (67), that genetically modified GFP-tagged Env (gp120) recycling can be detected.

Using this secondary staining recycling assay, we observed a pool of recycled Env near the PM suggesting exocytosis had occurred. Interestingly, a portion of recycled Env was detected in intracellular compartments, indicating that it had been reendocytosed, suggesting that recycled Env is not exclusively incorporated into assembly sites upon plasma membrane return. Notably, the levels of secondary staining varied greatly between cells, with a population of cells showing little or no secondary signal above background, even while harboring bright intracellular b12 Fab signal. This suggests that the rate of Env recycling is variable across the cell population, or alternatively, Env recycling is tightly regulated compared to Tfn in virus-infected T cells.

To further examine whether recycled Env might be incorporated in HIV-1 particles, the recycling assay was performed on cells expressing an FP-tagged nanobody probe for HIV-1 Gag (68). Some puncta of secondary signal colocalized with Gag at the PM (Fig. 7C), suggesting incorporation of recycled Env in Gag lattices. When the acid-stripping step was omitted, leaving b12 Fab bound to surface-exposed Env, much more robust secondary staining was observed (Fig. 7D), indicating that the amount of pulse-labeled Env that was internalized by the time of acid-stripping and then recycled to the PM was minor compared to the amount of pulse-labeled Env that was already present on the PM prior to stripping. Uninfected cells lacked both b12 Fab and secondary signal (Fig. 7E), confirming that the Fab labeling was specific to the expression of Env in infected cells. Overall, the results of this assay demonstrate that native endocytosed Env can return to the PM, where a minor fraction can be incorporated into virions.

### Endocytic Env does not readily mobilize to the virological synapse.

**FIG 8:**
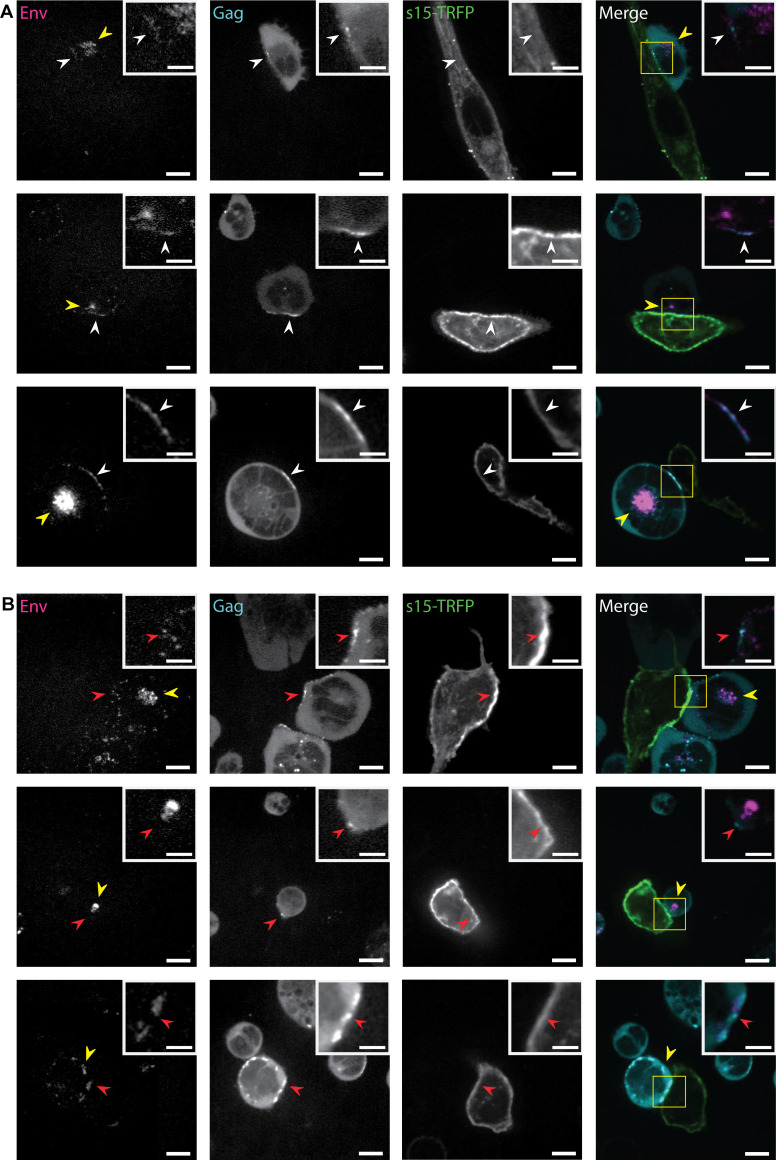
The total endocytic Env pool infrequently recycles to virological synapses between infected T cells and naive macrophage over minutes to hours. (A) Montage of HIV-1-infected CEM-A T cell virological synapses (Env, magenta; Gag, cyan) with naive differentiated THP-1 macrophages (green). CEM-A cells were pulse-labeled with anti-Env BG18 Fab AF647 probe (magenta) and chased for up to 3 h after coincubation with THP-1 macrophage. (B) CEM-A cells were pulse-chase labeled as above, except acid treated to remove surface exposed fab prior to chase and incubation with THP-1 macrophage (~3 h). Under acid-treated conditions, while proximity was observed between CEM-A produced Gag and THP-1 macrophage membranes, very little colocalization with endocytic Env was observed relative to the overall intracellular pool. We did observe, however, instances of endocytic Env in vesicular-like structures docked proximal to the PM. Collectively, these results suggest that steady-state PM labeling of Env probes, without prior acid stripping of Fab, highlights preexisting virus assembly site associated Env. Furthermore, once internalized, the majority of the endocytic Env pool remains perinuclear and infrequently recycles to cell-to-cell synapses under these experimental conditions. White arrows indicate colocalization between Env, Gag, and THP-1 s15-TagRFP, while red arrows indicate a lack of colocalization. Yellow arrows highlight the intracellular (endocytic) pool of Env that has not mobilized to the PM. Scale bars are 15 μm. Inset scale bars are at 5 μm.

**FIG 9:**
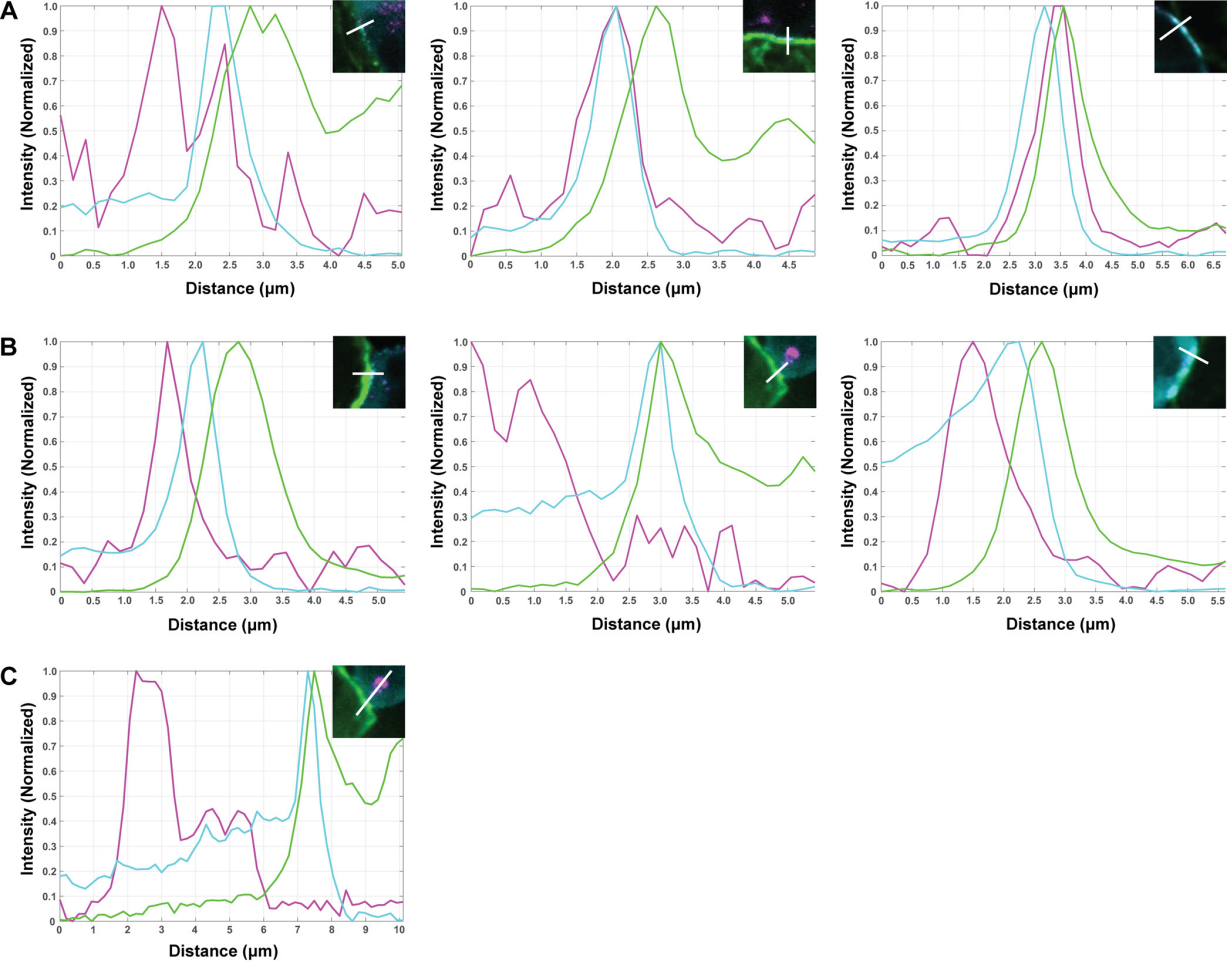
Line-scan analysis of HIV-1 Env signal enrichment at virological synapses between infected CEM-A T cells and naive THP-1 macrophage. (A) Line-scan of HIV-1 infected CEM-A cells with differentiated THP-1 macrophage (naive) from Fig. 8A, insets. The intensity of each channel was background subtracted and normalized for peak intensity in the line-scan region of interest. Overlap between Env labeled BG18-AF647 (magenta), CANTD-eGFP labeling Gag (cyan), and s15-TagRFP membrane marker on macrophage (green) was observed. (B) Acid-stripped infected CEM-A cells synapsing with THP-1 macrophage from Fig. 8B, insets. Overlap between the Gag (cyan) and THP-1 marker (green) was apparent, however, less overlap between these two channels and Env BG18-AF647 (magenta) was observed compared to non-acid-stripped measurements. (C) Line-scan of panel B center was extended and adjusted to include the intracellular pool of Env. Compared to the perinuclear intracellular pool of Env, Env that is VS-proximal is minimal, suggesting that the endocytic recycling to the VS does not readily occur under these experimental conditions.

As the majority of pulse-labeled Env was retained in intracellular compartments, we next sought to determine if recycling of Env cargo was regulated by physiological stimuli such as cell-to-cell contact. It is well documented that HIV-1-infected CD4^+^ T cells form cell-to-cell contacts that enrich for viral components and lead to direct virus transmission *in vitro* (69–72). These T-cell contact sites, referred to as virological synapses (VSs), possess PM domains enriched in HIV-1 Env/Gag and CD4 in the infected and naive target cell, respectively. We established a virological synapse assay by coculturing CEM-A T cells and differentiated THP-1 macrophage cells, a cell type also infected *in vivo* by HIV-1. We observed enrichment of Gag and Env at sites of cell contact between infected CEM-A T cells and naive THP-1 macrophages (Fig. 8A). The intracellular pool of endocytosed Env remained abundant in CEM-A T cells synapsing with THP-1 macrophages, suggesting that formation of a virological synapse, with this particular assay, does not result in triggered secretion of bulk endocytic pools of Env. Acid stripping of surface-exposed anti-Env BG18 Fab probe, prior to coculture with THP-1 macrophages, showed that virological synapses contained very little labeling for Env of endocytic origin (Fig. 8B). Furthermore, line scan analysis shows that endocytic Env appeared proximal, but often slightly adjacent, to sites of Gag enrichment at virological synapses (Fig. 9), suggesting that this population could represent docked vesicles of endocytic or exocytic origin. These results suggest that endocytic pools of Env are not triggered *en masse* to recycle and secrete at the virological synapse using this pulse-chase coculture system, yet strong virological synapsing was observed. Additionally, we performed reciprocal assays, where THP-1 macrophages were infected with HIV-1 and then cocultured with naive CEM-A T cells (Fig. S5A). We observed virtually identical phenotypes, where the intracellular pulse-labeled Env pool was not mobilized in bulk upon virological synapse formation, yet Gag and Env were enriched on the PM of infected THP-1 macrophages at sites of cell-to-cell contact with CEM-A cells. Additionally, acid stripping of pulse-labeled Env on infected THP-1 cells led to removal of surface exposed anti-Env BG18 Fab probe, and after relevant cellular migration chase times (0.5 to 2.5 h), very little endocytosed Env was observed at sites of cellular contact when compared to the signal from the overall intracellular pool (Fig. S5B). Collectively, these results suggest that the recycling of the Env endocytic pool may provide new trimers to the virological synapse between T cells and macrophages, but compared to the magnitude of the endocytic Env pool, this contribution is relatively small over relevant timescales for cellular interaction.

## DISCUSSION

The endocytosis of HIV-1 Env from the surface likely constitutes an important mechanism to limit antigen exposure on the surface of infected cells. In this study, we characterized the fate of HIV-1 Env upon endocytosis from the cell surface during virus assembly in infected CD4^+^ T cells. This postendocytic trafficking of Env has been controversial, with several different studies showing evidence for its transport to different compartments and via different host cell factors (31, 32, 34, 35) (Fig. 1).

We used several key experimental approaches to investigate these questions in a physiologically relevant context. First, the experiments were performed in CD4^+^ T-cell lines to more closely represent the main cell type targeted for infection by HIV-1. This is important considering that the role of Env gp41 CT is cell-type dependent (12) and that some potential trafficking pathways for endocytosed Env, namely, the secretory lysosome pathway (35), are cell-type specific. Next, CRISPR/Cas9-mediated endogenous protein tagging (Fig. S1) enabled us to image endogenously expressed Rab GTPases, avoiding the problems of Rab overexpression artifacts. Furthermore, pulse-chase assays using monovalent anti-Env Fab fragments enabled us to specifically label and image endocytosed native Env in HIV-infected cells, without perturbing trafficking by cross-linking Env trimers. Simultaneous pulse-labeling of Tfn was used to compare the trafficking of Env to that of a well-studied recycling cargo. Additionally, secondary staining against the anti-Env Fab allowed us to detect recycling and return of endocytosed Env to the PM. Finally, we utilized a coculture assay between T-cell and macrophage lines to assess the role of cell contact and virological synapse formation in regulating recycling and secretion of endocytic Env pools. Conversely, an important caveat to our approach is the reliance on the T-cell line-adapted (TCLA) HIV-1 strain NL4-3. Although TCLA HIV-1 strains such as NL4-3 are widely used for HIV-1 research, and one comparative study has reported that Env surface expression and proteolytic processing are similar for TCLA Env compared to primary HIV-1 isolates (73), some studies have indicated that natural polymorphisms in primary isolate Envs can alter cell-surface expression (74), intracellular trafficking, and virion incorporation (75) compared to TCLA Envs. Thus, future experiments with primary HIV-1 isolates will be important to confirm whether our findings are conserved across HIV-1 variants. A related caveat is that this study relied on immortalized CD4^+^ T-cell lines such as CEM-A rather than primary T cells, in part because the smaller diameter of primary T cells makes it difficult to resolve the various endocytic compartments by standard light microscopy imaging. Since different CD4^+^ T-cell lines may better represent primary CD4^+^ T cells in different aspects of HIV replication and intracellular trafficking (76–78), we have controlled for cell-line-specific effects by confirming key findings in multiple T-cell lines, but it will be important for future studies to examine Env trafficking to this level of detail in HIV-infected primary CD4^+^ T cells.

In this study, we assessed the trafficking of Env with respect to the Rab GTPase Rab5A, Rab11A, and Rab14, having known roles in endosomal regulation (Fig. 2). Upon internalization, Env as well as Tfn trafficked through the Rab5A^+^ EE, since they were observed to partially overlap with endogenous Rab5A and were trapped by a constitutively active Rab5A mutant. In contrast, we found that endocytosed Env did not localize to the Rab11A^+^ ERC, since it displayed little overlap with endogenous Rab11A and was not trapped by overexpressed Rab11A. This was a somewhat surprising result, because, both in this study and in previous work (37), internalized Env was observed to overlap with Tfn, which is known to recycle via the ERC (39, 42). However, our experiments demonstrate that Tfn could traffic by multiple pathways in these cells, including the Rab11A-mediated ERC pathway as well as the Rab11A-independent pathway taken by Env.

Previous work from the Spearman lab (31, 32) has concluded that endocytosed Env is trafficked to the ERC. Given that the ERC is molecularly defined by the presence of Rab11, our observations did not indicate Env localization in the ERC in T cells. The experiments that indicated Env trafficking to the ERC (31, 32), however, were performed in HeLa cells, suggesting that cell-type differences between trafficking pathways may lead to alternative routing of Env cargos when compared to T-cell lines. Another possible explanation for the routing to the ERC could be from the use of a chimeric Env construct with a synthetic ectodomain fused to the transmembrane and cytoplasmic tail regions of HIV-1 Env. It is possible that this synthetic Env mimic traffics differently when compared to the native full-length Env trimers used in our experiments. These studies also did not find that Env recycling was dependent on Rab11 *per se*, but rather on Rab14 and on Rab11-family interacting protein 1C (FIP1C), which is a shared effector for both Rab11 and Rab14 (31, 32). Our experiments showed that endocytosed Env, along with Tfn, overlapped substantially with endogenous Rab14. Staining with a quaternary-specific anti-Env Fab confirmed that this was the case for mature functional Env trimers (Fig. S2A). Collectively, our findings support a role for Rab14 in the trafficking of endocytosed Env, in the physiologically relevant context of full-length, mature trimeric Env in HIV-1-infected CD4^+^ T-cell lines.

We also assessed the localization of Env with respect to the Golgi/TGN and to LEs/lysosomes (Fig. 3). We found very little overlap of endocytosed Env with a Golgi/TGN probe. Previous studies (33, 34) have indicated that endocytosed Env undergoes retrograde trafficking from endosomes to the Golgi or TGN. Pulse-labeling to show the localization of endocytosed Env in these studies was performed in HeLa cells, and either labeled Env with a bivalent antibody or used a CD8-gp41 chimera, suggesting that the aforementioned potential cell-type differences and issues with these approaches for tracking Env could again be reasons for discrepancies between our observations. Our results also do not exclude the possibility that endocytosed Env retrograde traffics through the Golgi/TGN rapidly and therefore would not result in significant steady-state accumulation in these compartments. The retromer complex mediates retrograde trafficking to the Golgi/TGN, yet has also been implicated in functions for other endosomal recycling pathways independent of retrograde traffic to the Golgi/TGN (61). It is possible that Env interactions with retromer (34) are necessary for aspects of the Rab14-mediated and CD4^+^ T-cell-specific Env trafficking pathways observed in our studies.

In contrast to the proposed recycling pathways for Env, we observed that endocytosed Env overlapped significantly with LAMP1, which localizes mainly to LEs and lysosomes. This was also confirmed to be the case for mature Env trimers (PGT151 probe labeling) and in the SupT1 suspension T-cell line (Fig. S2). Env also colocalized with CTLA4 (Fig. 3), which traffics to lysosomes where it can either be degraded or be secreted by a secretory lysosome pathway in T cells (52). LysoTracker staining demonstrated that many of these LAMP1^+^ and CTLA4^+^ compartments were acidic organelles. Our results, therefore, show trafficking of endocytosed Env to LEs and lysosomes in T cells. These observations support the hypothesis that endocytosed Env traffics to the secretory lysosome pathway (35); however, this is also consistent with Env trafficking to lysosomes for degradation. Additional nondegradative trafficking pathways out of the LE or lysosome may also exist; for example, a lysosome-endosome-Golgi retrograde pathway has been demonstrated in yeast (79). Interestingly, depletion of Rab7A, which regulates late endosomal and lysosomal trafficking, was previously shown to impair HIV-1 infectivity in part by disrupting Env maturation (proteolytic processing of the Env gp160 precursor into gp120 and gp41) (80), suggesting the possibility that Env trafficking through LEs and/or lysosomes may regulate the frequency of encounter with furin-like proteases.

We furthermore found that LEs and lysosomes, as well as EEs, were the main compartments to which Rab14 localized in T-cell lines (Fig. 4). This may indicate an uncharacterized and likely cell-type-specific role for Rab14. In HeLa and NRK epithelial cell lines, Rab14 localizes to EEs and to the Golgi and is proposed to function in trafficking between these compartments. In these studies, Rab14 did not colocalize with lysosome markers (47), suggesting the role of Rab14 in epithelial cells may be distinct from that of T cells. In the protist *Dictyostelium*, the lysosome is a secretory organelle rather than a terminal degradative organelle, and the Rab14 orthologue localizes to lysosomes, functioning in lysosomal fusion (81). It is therefore possible that Rab14 functions in lysosomal trafficking specifically in cells where lysosomes act as secretory granules. We additionally showed that a constitutively active Rab14 mutant colocalized with Env in EEs and LEs/lysosomes and reduced cell-surface levels of Env (Fig. S3). Together with previous work from the Spearman lab, which showed that mutation or depletion of Rab14 in HeLa cells perturbed virion incorporation of Env (31), these results support a role for Rab14 in regulating Env trafficking *en route* to virion incorporation.

A biochemical analysis of enriched lysosomal fractions from HIV-infected cells confirmed that Env and Fab probes are transported to lysosome-like compartments after its internalization from the PM (Fig. 5 and Fig. S4). Since trafficking to LEs and lysosomes is most often a degradative pathway, we additionally examined whether endocytosed Env was degraded (Fig. 6). We observed relatively little loss of Env over 5 h, suggesting that even though Env trafficked to LEs and/or lysosomes, it was not rapidly degraded there. For comparison, around half of endocytosed CTLA4 in T cells has been reported to undergo lysosomal degradation within 3 h (66), suggesting that the persistence of internalized Env in this assay was not simply due to the time being too short for degradation to occur. In contrast, clear loss of internalized Tfn was observed over time; however, the rate of this decrease was relatively slow, showing a half-life roughly on the order of an hour, compared to reported rates for Tfn recycling, including in T cells, with a *t*_1/2_ ≈ 10 min (82). This may indicate that Tfn recycling by the constitutive recycling pathways was a relatively small portion of the Tfn pool in our assays, explaining why the majority of Tfn signal colocalized with Env in LEs and lysosomes (Fig. 3) rather than its expected localization in the EE and ERC. It is possible that the decrease we measured for Tfn mainly represents either lysosomal degradation of Tfn receptor (TfR) (83, 84) or return to the cell surface by a unique slower pathway induced by HIV-1 infection and shared with Env. TfR recycling is constitutive and passive, occurring by bulk membrane flow, and this pathway is not dependent on the cytoplasmic domain of TfR (85, 86). However, TfR can also traverse specific polarized secretion pathways in polarized cells. For example, TfR is selectively trafficked to the basolateral membrane in MDCK cells (87) and to the immunological synapse (IS) in T cells (88). HIV-1 Env is also targeted to the basolateral membrane in MDCK cells (89) and to the T-cell VS, which bears important similarities to the IS (35, 90). It is therefore plausible that endocytosed Tfn can take a specific trafficking pathway, also taken by endocytosed Env, and this Tfn pool might be more prominent than the rapid constitutive recycling pool of Tfn during HIV-1 infection.

Next, we tested whether Env physically recycles to the cell surface after endocytosis (Fig. 7), as this has not previously been demonstrated for native Env trimers, although a very recent study (67) detected recycling of an Env-GFP fusion protein. Here, we demonstrate evidence of endocytosed native HIV-1 Env having returned to the PM (Fig. 7), which is a prerequisite for its incorporation into HIV-1 assembly sites. Much of the recycled Env detected was intracellular, showing that it had been reinternalized rather than incorporated in virus, but some puncta of recycled Env signal colocalized with Gag at the PM, suggesting it is possible for recycled Env to incorporate into viral assembly sites. It is important to note, however, many cells showed little to no signal of recycled Env, suggesting that the constitutive rate of Env recycling, if any, was relatively low. One possible explanation for the variability in the rate of Env recycling would be that endocytosed Env returns to the PM, not by constitutive recycling, but rather by a regulated secretion process. This regulated secretion pathway could necessitate specific conditions or stimuli to trigger secretion and may explain why only a fraction of cells in these assays displayed recycled Env signal. Regulated secretion of transmembrane proteins in CD4^+^ T cells is believed to occur via secretory lysosomes (91), so the secretory lysosome mechanism is a likely candidate for such a process, as proposed by Jolly et al. (35), although that study found that Env secretion by this mechanism occurred in response to VS formation, and it is currently not clear what stimulus induces recycling/secretion in the absence of cell-cell contacts. Overall, the cell-type specificity of the secretory lysosome pathway may explain why other studies using HeLa cells have reached distinct conclusions about endocytic Env trafficking and recycling pathways.

Subsequently, we sought to determine the contribution of recycled Env in enriching the VS and whether the endocytic pool of Env could be triggered for secretion via cell-to-cell contact. We established a coculture assay between THP-1 macrophages and CEM-A T-cell lines, two cell types infected *in vivo* by HIV-1. Infected CEM-A T cells formed VS contact sites with naive THP-1 macrophages that were enriched in Gag and Env (Fig. 8). Reciprocal experiments where THP-1 macrophages were infected with HIV-1 displayed similar VS morphologies when cocultured with CEM-A T cells (Fig. S5). Under both experimental culture conditions, pulse-labeled Env was observed at the VS and in intracellular compartments; however, cell-to-cell contacts did not result in bulk secretion of endocytic Env to the PM, suggesting that VS formation itself may be insufficient for mobilizing this pool of Env or conversely, the majority of this pool is not associated with the regulated secretory pathway. Under these experimental conditions, we observed that the endocytic pool of Env can contribute to VS enrichment of Env hours after cell-to-cell contact. This fraction of Env appeared to dock at the plasma membrane in vesicular pools but was observed to be a relatively minor fraction of the overall endocytic pool, suggesting that newly biosynthesized and existing plasma membrane Env pools, through lateral diffusion on the PM, may contribute to the bulk of Env enriched at the VS. Indeed, our previous work demonstrated that Env diffusion along the PM is one mechanism for locating budding virus assembly sites and creating infectious particles (15). Our findings are consistent with those of a recent study (67) that found, in T cells infected with an HIV-1 clone carrying an Env-GFP fusion, that recycling of endocytosed Env to the VS occurred but was not required for Env accumulation at the VS. Since the genetic tagging of Env, even in the optimized manner used in that study, perturbs Env function as evidenced by reduced virus infectivity and slower spread of infection (67), our results, representing recycling of wild-type Env, importantly show that this is not an artifact of the tagging approach. These results suggest that the endocytic pool of Env in CD4^+^ T cells could be a storage mechanism that can prevent excess display of viral epitopes. Indeed, it has been suggested that endocytosis of excess Env may reduce the likelihood of antibody-dependent cellular cytotoxicity (ADCC) and assist immune evasion (3, 4, 36, 92). It is important to note, however, that an ADCC evasion mechanism is not mutually exclusive for the role of Env recycling in creating infectious particles on the PM.

**FIG 10:**
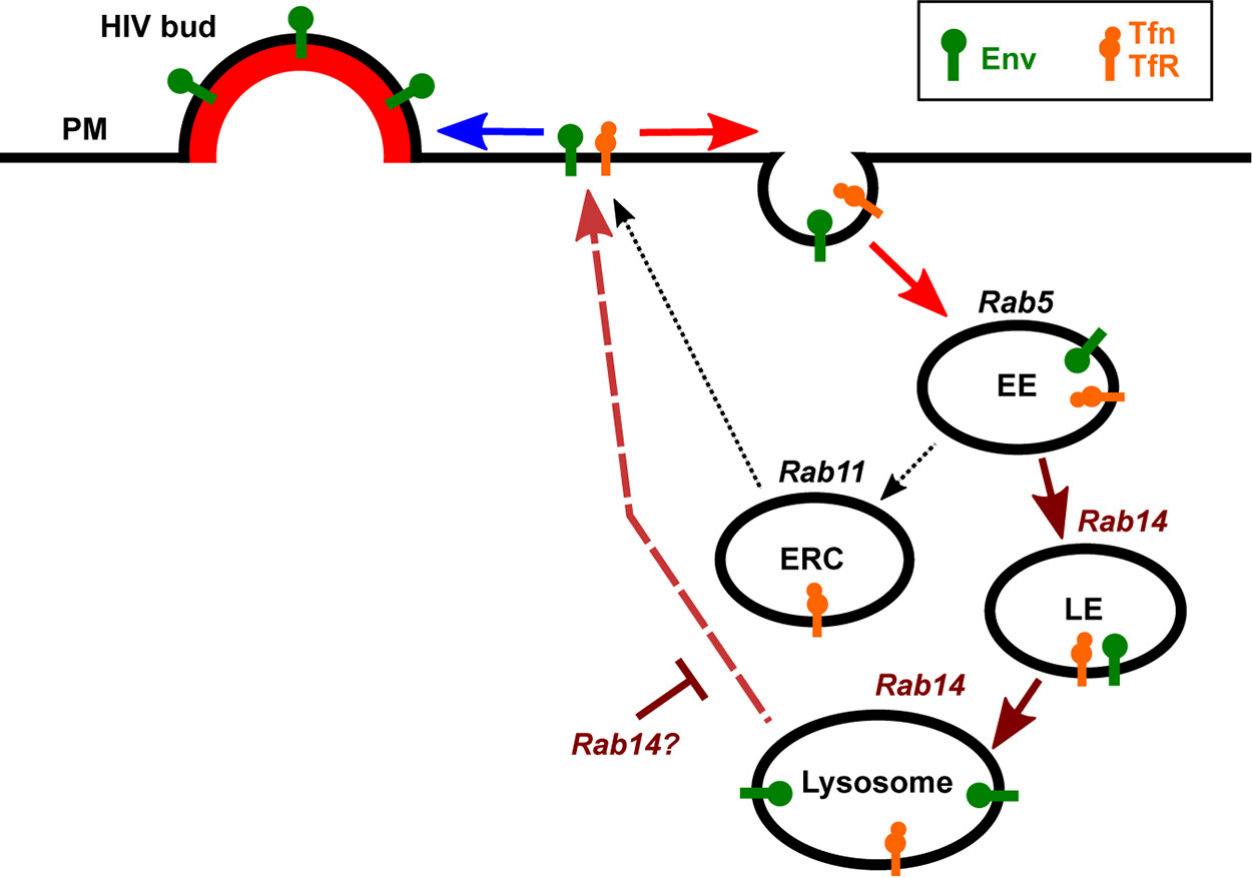
Revised model of endocytic HIV-1 envelope glycoprotein trafficking routes. HIV-1 Env trimers which fail to encounter a virus assembly site on the PM are endocytosed. Upon PM internalization, both Env and the model recycling cargo, Tfn, are delivered to Rab5^+^ early endosomes (EE). Env is then sorted, potentially through trafficking intermediates in the endocytic pathway, into Rab14^+^ LE and lysosomes, which possess LAMP-1, low pH (Lysotracker labeling), and the secretory lysosome marker CTLA4 (not shown). A population of Tfn cotraffics along this endosomal route with Env, while a fraction of the Tfn pool recycles via the canonical Rab11^+^ ERC. Env is retained in LE/lysosomal compartments mostly without being degraded and appears to be infrequently recycled and secreted back to the PM where it may be incorporated in virions. Retrograde trafficking pathways via the LE to trans-Golgi network (TGN) are possible; however, this retrograde pathway must be rapid, leading to very low levels of accumulation in Golgi type compartments at steady-state. Rab14 appears either to promote Env transport to lysosomes or to negatively regulate Env return to the PM. Collectively, the endocytic trafficking of HIV-1 Env may serve to both sequester excess viral epitope display on the PM and provide a route for recycling back to the PM to locate a virus assembly site and create an infectious particle. TfR, Tfn receptor.

Overall, our results collectively suggest a refined model for the postendocytic trafficking of HIV-1 Env in CD4^+^ T cells (Fig. 10). Following endocytosis from the cell surface, both Env and Tfn are delivered to the EE, which is regulated by Rab5. Env then traffics to Rab14-regulated compartments with characteristics of LEs and secretory lysosomes. A portion of Tfn cotraffics along this route with Env, while another portion of Tfn recycles via a canonical ERC-mediated route via Rab11. Env is retained in LE/lysosomal compartments over a period of hours, mostly without being degraded. Finally, this internalized Env is infrequently secreted back to the PM where it may be incorporated in virions.

This revised model raises several important questions to be addressed by future studies. First, we have shown that Rab14 in CD4^+^ T cells is associated with LEs and lysosomes, but the function of Rab14 on these compartments remains to be determined. Ongoing experiments to track endocytosed Env and other cargos in the presence of Rab14 mutants or Rab14 depletion could reveal whether Rab14 is responsible for cargo delivery to LEs/lysosomes, or for regulating secretion of lysosomes, or some unidentified function. Additional detailed characterization of host-cell trafficking dependencies will inform whether targeting the postendocytic pathway could be a useful antiretroviral treatment strategy, with the potential either to prevent Env incorporation and render virus particles noninfectious or to reciprocally increase Env surface exposure and recruit a protective ADCC immune response against HIV-1.

## MATERIALS AND METHODS

### Cell lines and culture conditions.

The CEM-A cell line (CEM-CL10) (38), an adherent CD4^+^ T-cell line, was obtained from Dr. Mark Wainberg and Dr. James McMahon through the NIH AIDS Reagent Program (Germantown, MD). The P2 cell line, a polarized T-cell line derived from a clone of the A3.01 T-cell line (53), and the SupT1 T-cell line (14, 51), were gifts from Dr. Eric Freed. The HEK293T cell line used for lentivirus production was obtained from ATCC (Manassas, VA). Cells were cultured in complete growth medium at 37°C with 5% CO_2_. Cell culture reagents were obtained from Corning, Inc. (Corning, NY). Complete growth medium contained 10% fetal bovine serum (no. 35-011-CV), 2 mM l-glutamine (no. 25-005-CI), and 1× penicillin-streptomycin (no. 30-002-CI) in RPMI medium (no. 17-105-CV) with 1× HT (hypoxanthine, thymidine) (no. 25-047-CI) for T cells or in DMEM (no. 17-205-CV) for HEK293T cells. CEM-A cells were passaged using Cellstripper solution to dissociate cells from culture dishes (no. 25-056-Cl) as described in the original derivation of the CEM-A cell line (38).

### Plasmids.

**(i) HIV-1 expression constructs.** The modified HIV-1 NL4-3 reference genome has been previously described (37). Briefly, the HIV-1 expression constructs used in this study contained the following modifications to the HIV-1 NL4-3 reference genome: (i) the p6 PTAP motif was mutated (455PTAP458-LIRL) (93) to prevent virus particle release, critical to avoid virus particles from being released from infected cells and taken up by other cells in the sample, thus ensuring that Env signal observed on or in a cell was produced within that cell; (ii) the *pol* gene was deleted by removal of the BclI-NsiI fragment; (iii) deletion of the *vif* and *vpr* genes via removal of the AflII-AflII fragment; and (iv) constructs expressing fluorescent probes, the *nef* gene was replaced with coding sequences for the probes, described below, using Gibson assembly (94). Because the FP-tagged probe is expressed in place of *nef* under the control of the HIV-1 5′-long terminal repeat (LTR) promoter, this strategy is useful to synchronize the timing of transient expression of the probe with the timing of virus assembly in infected cells. These modified HIV-1 NL4-3 genomes are denoted in this article as “pSV-ΔΔΔ-[probe]-3′LTR.” All experiments not relying on viral expression of FP-tagged probes (endogenous Rab tagging, etc.) possessed an intact *nef* coding region.

**(ii) Cloned fluorescent probes.** For GFP-Rab5A(Q79L), the Rab5A coding sequence was synthesized by Integrated DNA Technologies (IDT; Coralville, IA) and cloned into the Duet011 plasmid (no. 17627; Addgene, Watertown, MA) in-frame with GFP, and the Q79L mutation was introduced by site-directed mutagenesis. The Golgi/TGN probe SiT-GFP consisted of the N-terminal 45 residues of beta-galactoside alpha-2,6-sialyltransferase 1, a portion that lacks the enzyme catalytic domain but is sufficient for localization to the Golgi and TGN (95), fused to GFP (a gift from the Jennifer Lippincott-Schwartz laboratory). LAMP1-Emerald, consisting of *R. norvegicus* LAMP1 fused at its C-terminus to mEmerald, was obtained from Addgene plasmid no. 54149 contributed by Dr. Michael Davidson. For CTLA4-GFP, the CTLA4 coding sequence was synthesized by IDT and cloned into the pLJM1-EGFP plasmid (no. 19319; Addgene) in-frame with GFP. For the membrane marker s15-GFP (96), pEGFP-N1 was used as the template for GFP, and the membrane-anchoring signal of Src (amino acid sequence: *MGSSKSKPKDPSQRRNNN*) was appended into the 5′-PCR primer in-frame with the GPF coding sequence. CANTD-TagRFP, in which CANTD is a single-domain antibody (nanobody) that binds the CA domain of HIV-1 Gag (68), was derived from the pCANTD-EGFP plasmid, which was a gift from Heinrich Leonhardt (68). TagRFP (97) was cloned into the pCANTD-EGFP plasmid in place of EGFP. The Emerald-Rab14(Q70L) coding sequence was synthesized by IDT.

**(iii) Lentiviral transfer plasmids used to generate stable cell lines.** The lentiviral transfer plasmid pLEX-TagRFP-Rab11, encoding human Rab11A, was a gift from Dr. Erich Kushner (University of Denver). For the lentiviral transfer plasmid pLJM1-TagRFP-Rab14, a pLJM1-TagRFP vector was first generated by replacing the EGFP coding sequence in pLJM1-EGFP (no. 19319; Addgene) with a TagRFP coding sequence. The human Rab14 coding sequence was synthesized by IDT and cloned into this vector in-frame with TagRFP.

### Virus production.

Replication-incompetent, single-round infectious viruses were produced by cotransfecting HEK293T cells with the described lentiviral transfer plasmid (the modified HIV-1 NL4-3 expression constructs detailed in Plasmids above), psPAX2 packaging plasmid (a gift from Didier Trono, plasmid no. 12260; Addgene), and pVSV-G pseudotyping plasmid (a gift from Dr. Xuedong Liu, University of Colorado, Boulder), using polyethyleneimine (no. 43896; Alfa Aesar/Thermo Fisher Scientific, Tewksbury, MA). Virus was harvested approximately 48 h posttransfection, 0.45-μm filtered, and in most cases snap-frozen in liquid nitrogen and stored at −80°C until use. In a few instances, including the recycling assay, the virus was used for infection immediately without freezing in order to minimize loss of viral titer.

### Generation of TagRFP-Rab11 and TagRFP-Rab14 stable CEM-A lines.

Cell lines stably overexpressing TagRFP-Rab11 or TagRFP-Rab14 were generated by transducing CEM-A cells with lentivirus generated with pLEX-TagRFP-Rab11 or pLJM1-TagRFP-Rab14 respectively, pSPAX2 packaging vector, and pVSV-G, followed by selection with 500 ng·mL^−1^ puromycin.

### Production and dye-conjugation of anti-Env Fab probes.

The anti-Env b12 Fab recombinant expression vector pCOMB3H-b12 (55, 98) was a generous gift from Dr. Dennis Burton. The *pIII* gene was deleted from this vector by removal of the SpeI/NheI fragment, and a 4× lysine tag was added to the C-terminus of the b12 light chain. b12 Fab was expressed in E. coli XL1 Blue competent cells (Stratagene, San Diego, CA) as previously described (99). To purify b12, bacterial cell pellets were resuspended in PBS pH 7.4 with 0.2 mM PMSF (no. P-470-10; GoldBio, St. Louis, MO) and lysed by sonication. Clarified cell lysates were purified by protein G affinity chromatography (no. P-430-5; GoldBio), and the eluates were dialyzed overnight in PBS pH 7.4 at 4°C. The b12 Fab was then conjugated with Atto565 NHS ester (no. 72464; Sigma-Aldrich, St. Louis, MO) or Alexa Fluor 647 NHS ester (no. A20006; Thermofisher).

For the anti-Env Fabs PGT145 (22, 100), BG18 (56, 57), and PGT151 (46), the coding sequences for the heavy and light chains were synthesized by IDT and cloned into the pCOMB3H-b12 vector in place of the heavy and light chains of b12. PGT145, BG18, and PGT151 Fabs were expressed and dye-conjugated in the same manner as b12 Fab, except purification was accomplished using CH1-XL affinity resin (no. 2943452010; Thermo Fisher Scientific).

### Fluorescent protein Rab Cas9 knock-in.

CEM-A T-cell lines with endogenously tagged Rab5A, Rab11A, or Rab14 were generated by coelectroporation of CRISPR/Cas9 ribonucleoproteins (RNPs) and linear double-stranded DNA homology-directed repair template (HDRT), based on the method of Roth et al. (44).

For Rab11A, the guide RNA (gRNA) sequence validated by Roth et al. (44) was used, that is *GGTAGTCGTACTCGTCGTCG*. For Rab5A and Rab14, gRNA sequences were selected by using the CRISPOR gRNA design tool (101) to identify the gRNAs with the highest specificity and efficiency and lowest probability of off-target effects within a range of ~30 nucleotides of the start codons of the respective genes; the gRNAs for Rab5A and Rab14, respectively, were *AATTTGGACATGGCTAGTCG* and *GTATGGTGCAGTTGCCATGG*. To produce the gRNAs, the crRNA and tracrRNA components were synthesized by Thermo Fisher Scientific (Waltham, MA) and were annealed according to the manufacturer’s protocol.

The HDRT sequence for each KI consisted of the FP coding sequence superfolder GFP (102) for Rab11A and Rab14, or mRuby3 (103) for Rab5A, flanked by 350- to 400-bp homology arms for the genomic regions upstream (5’UTR of the Rab) and downstream (Rab exon 1 in-frame with the FP, and a portion of intron sequence). The GFP-Rab11A HDRT source plasmid from Roth et al. (44) was obtained from Addgene (no. 112012). The mRuby3-Rab5A and GFP-Rab14 HDRTs were synthesized by IDT. Each HDRT was amplified by PCR and gel purified.

Electroporation was performed with the Neon Transfection System (no. MPK5000) and Cas9 protein (no. A36497) from Thermo Fisher Scientific. Approximately 5 × 10^5^ CEM-A cells were electroporated with 25 pmol of RNPs (1:1 Cas9:gRNA molar ratio) and 2 μg of HDRT using the Neon Transfection System 100 μL Kit (no. MPK10096) according to the manufacturer’s protocol, with pulse conditions of 1,700 V per 20 ms per pulse. The cells were then expanded in culture, and GFP^+^ or mRuby3^+^ cells were isolated 1–2 weeks after electroporation by fluorescence-activated cell sorting. The percentages of positive cells for mRuby3-Rab5A KI, GFP-Rab11A KI, and GFP-Rab14 KI CEM-A were 4.73%, 10.7%, and 19.4% respectively.

### Western blots of fluorescent protein Rab GTPase knock-in CEM-A lines.

For each Western blot sample, 6 × 10^6^ cells were washed with DPBS, pelleted, and stored at −80°C. Cell pellets were thawed and lysed on ice in 400 μL of ice-cold RIPA buffer (50 mM Tris, pH 7.5, 150 mM NaCl, 1% Triton X-100, 1 mM EDTA) with protease inhibitor cocktail (no. S8830; Sigma-Aldrich) for 30 min and then clarified by centrifugation at 10,000 × *g* for 20 min at 4°C. The cell lysates were normalized for total protein concentration by BCA assay (no. 23227; Thermo Fisher Scientific). Proteins were resolved by SDS-PAGE on 4–20% gradient gels (no. 4568096; Bio-Rad, Hercules, CA) and transferred to nitrocellulose membranes.

Rab5 Western blotting was accomplished by blocking the membrane with 5% (wt/vol) nonfat dry milk in Tris-buffered saline pH 7.5 with 0.1% Tween (TBST-M) and then probed with rabbit monoclonal anti-Rab5 antibody [EPR21801] (no. ab218624; Abcam, Cambridge, UK) at 1:1,000 in TBST with 5% milk overnight at 4°C. The blot was then with TBST, stained with horseradish peroxidase (HRP) anti-rabbit secondary antibody (Invitrogen) at 1:10,000 in TBST with 5% milk for 1 h at room temperature (RT) and washed with TBST.

Rab11 Western blotting was performed as follows: the membrane was blocked with TBST-M, washed with TBST, and then stained with rabbit polyclonal anti-Rab11 antibody (no. 3539; Cell Signaling Technology, Danvers, MA) at 1:1,000 in TBST with 5% BSA overnight at 4°C. This antibody recognizes both Rab11A and Rab11B isoforms. The blot was then washed with TBST, stained with HRP anti-rabbit secondary antibody (Invitrogen) at 1:10,000 in TBST-M 1 h at RT, and washed with TBST.

Rab14 Western blotting was performed by blocking the membrane with TBS-M (without Tween) and stained with mouse monoclonal anti-Rab14 antibody (D-5) (no. sc-271401; Santa Cruz Biotechnology, Dallas, TX) at 1:100 in TBST-M overnight at 4°C. The blot was then washed with TBST, stained with HRP anti-mouse secondary antibody (Invitrogen) at 1:6,000 in TBST-M for 1 h at RT, and finally washed with TBST.

For each blot, the secondary HRP conjugates were detected using ProSignal Dura ECL Reagent (no. 84-834; Genesee Scientific, San Diego, CA), and blot images were acquired with a FluorChem imager (ProteinSimple, San Jose, CA). Membranes were then reblocked and probed with mouse anti-α-Tubulin (clone B-5-1-2; no. T5168; Sigma) labeled with NHS-Alexa Fluor 647 (Invitrogen) for a loading control.

### Endocytic pulse-chase assay.

Pulse-chase assays to label surface-exposed and endocytosed Env were performed as follows. For the adherent T-cell line CEM-A, cells were seeded onto an 18-mm glass coverslip (sterilized by soaking in ethanol and air-dried prior to use) in a 3.5-cm dish, to be subconfluent at the time of infection. For the suspension P2 T-cell lines, approximately 0.6 to 1.0 × 10^6^ cells were seeded into a 3.5-cm dish. Each sample was infected with 1 mL of virus, which was produced as described under Virus Production above, at 40 to 45 h prior to staining.

Cells were blocked for 30 min at 37°C in media (complete RPMI media with HT; see above) with 10% BSA (no. A3983; Sigma-Aldrich) and then stained with the indicated probes in media with 6% BSA. Both CEM-A and P2 cells were stained for 15 min at 4°C, except where specified otherwise Fabs b12-AF647 or b12-Atto565 (produced in-house; see above) was used at 20 nM. Tfn-AF647, Tfn-AF55, or Tfn-AF488 (no. T23366, T35352, or T13342; Invitrogen) were used at 25 μg·mL^−1^. After staining, cells were washed 3 × 5 min with media at 37°C and kept incubating in the third wash at 37°C until the end of the indicated chase time (1 h for the assays on CEM-A and P2, except where specified otherwise). For assays with LysoTracker, LysoTracker Red DND-99 (no. L7528; Invitrogen) was included in the third wash at 1:2,000 dilution. The cells were fixed for 30 min with 4% paraformaldehyde (no. 15710; Electron Microscopy Sciences, Hatfield, PA) and 0.2% glutaraldehyde (no. 16220; Electron Microscopy Sciences) in DPBS, quenched with 3 × 5 min washes of 30 mM glycine (no. G8898; Sigma-Aldrich) in DPBS, mounted on slides with Fluoromount-G (no. 0100-01; Southern Biotech), and sealed with clear nail polish.

For CEM-A pulse-chase assays, the blocking and chase incubations were performed in a CO_2_ incubator at 5% CO_2_. For suspension cells, an 18 mm coverslip in a 3.5 cm dish was precoated with 100 μL of 0.1% (wt/vol) poly-l-lysine (Ted Pella Inc, Redding, CA; no. 18026) for 5 min, rinsed with 2 mL of sterile H_2_O, and air-dried prior to the assay. The blocking, staining, and chase steps were performed on the suspension cells in a 15-mL conical tube, which was centrifuged at 500 × *g* for the final 5 min of each incubation period to pellet the cells. Prior to fixation, the cells were resuspended in 100 μL of media, seeded onto the poly-l-lysine coated coverslip, and allowed to settle for 10 min; fixation and quenching steps were then performed on the coverslip in the 3.5 cm dish.

For assays with an acid-stripping step, the cells were placed on ice after staining, washed briefly with ice-cold DPBS, and stripped for 1 min with ice-cold acid-stripping buffer (0.2 M acetic acid, 0.5 M NaCl, pH ~3, in sterile ddH_2_O). The cells were then washed briefly several times with ice-cold DPBS to return to physiological pH (7.4).

### Recycling assay.

For the recycling assay, the infection, blocking, and primary staining steps were similar to the pulse-chase assay, except that the cells were stained for 20 min at 37°C.

C with 5% CO_2_. After staining, acid-stripping was performed by placing the samples on ice, washing for 1 min with ice-cold DPBS, and stripping for 1 min with chilled acid-stripping buffer. The cells were then washed 2 × 1 min with ice-cold DPBS, reblocked for 3 min in complete RPMI media supplemented with 10% BSA, and then stained with Alexa Fluor 488-labeled mouse anti-human kappa light chain secondary antibody (no. MH10520; Invitrogen) at 1:25 dilution for 1 h at 37°C with 5% CO_2_. For the assays utilizing DRAQ7, the DRAQ7 dye (no. D15105; Invitrogen) was included in the secondary stain at 1:100 dilution. The samples were then fixed with 4% paraformaldehyde and 0.2% glutaraldehyde and mounted on slides identical to the pulse-chase assay.

### Confocal fluorescence microscopy.

Confocal imaging was performed with a customized inverted Nikon Ti-E microscope (Solamere Technology Group Inc., Salt Lake City, UT) using a 60× CFI Plan Apo Lambda 1.4 NA oil-immersion objective (Nikon Instruments; Melville, NY). Fiber-coupled lasers (OBIS CW solid-state lasers, Coherent; Santa Clara, CA) were used in combination with a CSU-X A1 spinning disk unit (Yokogawa Electronics; Tokyo, Japan) to excite and collect confocal fluorescence sections. Z-slices were collected at 0.3-μm spacing.

### Immunofluorescence for total surface expression of Env.

For the Env surface-staining experiments shown in Fig. S3G and H, WT CEM-A T cells were infected with single-round infectious lentivirus produced from NL4-3 HIV-1, with deletions in *pol*, *vif*, *vpr*, *nef*, and the p6 domain as described above. Control conditions relied on expression of the CANTD-EGFP nanobody (described above) from the *nef* splice site. For infection conditions with coexpression of Rab14(Q70L) virus, pSV-ΔΔΔ-Rab14(Q70L)-3′LTR, the *nef* coding region was replaced with mEmerald-Rab14(Q70L). After 48 h postinfection, cells were live stained with Cell Mask Orange according to the manufacturer protocol (C10045; Thermo Fisher), washed to remove excess dye, and then fixed with 4% paraformaldehyde. The fixed but unpermeabilized cells were blocked in 10% BSA in DPBS for 30 min and stained (~20 nM) with the full IgG antibody anti-gp120 b12 directly conjugated to AF647 for 15 min at RT. Cells were washed 3 × 5 min in DPBS and immediately imaged on a Nikon spinning disk confocal. The coverslip-proximal plasma membrane was imaged exclusively for measurements of ectopic Env. Cells were then segmented manually in FIJI (104) using the Cell Mask channel only. Total surface expression of Env, as represented by b12-AF647 fluorescence density, was quantified using a custom MATLAB (MathWorks) software package. Fluorescence was normalized across samples to the mean fluorescence density of Env in control cells, and statistical significance was determined using the MATLAB ttest2 function.

### Lysosome isolation and biochemical analysis.

For the lysosome biochemistry experiments, CEM-A cells were seeded in two 182-cm^2^ tissue culture flasks (CT-229351; CellTreat) and infected between 60 and 80% confluence. Each flask was infected with 10 to 12 mL of virus, which was produced as described above in Virus production using the modified HIV-1 expression construct pSV-ΔΔΔ-3′LTRX. At ~44 h postinfection, the live cells were blocked in media with 10% BSA for 1 h, then stained with anti-Env Fab BG18 at 3.6 nM in media with 6% BSA for 20 min, washed 3 × 13 min with media and once briefly with DPBS, followed by dissociation from the flask using Cellstripper (Corning) for a total chase time of 1.25 h. Cells were maintained at 37°C and 5% CO_2_ throughout these blocking, staining, and wash steps. Collected cells were washed twice with ice-cold DPBS. A control sample of the cells prior to lysosomal isolation was taken at this point and lysed using RIPA buffer (described above, but containing 2% TX-100 to solubilize membranes).

Lysosomal enrichment and isolation were performed using reagents from the Lysosomal isolation kit (ab234047; Abcam). Cells were resuspended in lysosome isolation buffer containing Protease Inhibitor Cocktail and vortexed for 5 sec followed by a 2-min incubation at 4°C. Cells were then homogenized using a Dounce homogenizer with 30× passes using a tight pestle on ice. An equal volume of Lysosomal Enrichment Buffer was then added to the sample, which was mixed by inversion and centrifuged for 10 min at 500 × *g* and 4°C, and the supernatant containing isolated lysosomes was collected. Lysosomes were further enriched by discontinuous density gradient centrifugation following the manufacturer protocol. Ultracentrifugation was performed at 145,000 × *g* for 2 h at 4°C using a Beckman Coulter Optima MAX-XP Ultracentrifuge and TLS-55 rotor. Following ultracentrifugation, serial fractions were taken by hand from the top of the gradient. Fractions were further purified via precipitation by mixing the fraction with two volumes of cold DPBS and centrifugation at 18,000 × *g* for 30 min at 4°C. Pellets were then resuspended in DPBS and prepared for Western blotting analysis.

For Western blotting of enriched lysosomal fractions, proteins were resolved by SDS-PAGE on 4–20% gradient gels (no. 4568096; Bio-Rad, Hercules, CA) and transferred onto a 0.45 μm PVDF membrane (no. 83-646R; Genesee Scientific, San Diego, CA). The membrane was blocked with 5% (wt/vol) nonfat dry milk in PBS pH 7.4 with 0.1% TWEEN-20 (PBST-M) for 1 h at RT prior to each primary antibody staining. The following primary antibody conditions were utilized in 5% PBST-M in the following order: 1:1,000 mouse monoclonal [JDC-12] anti-human λ light chain HRP (no. ab99811; Abcam, Cambridge, UK), overnight at 4°C; 1:1,000 mouse anti-gp41 Chessie 8 (no. ARP-13049; NIH HIV Reagent Program), overnight at 4°C; 1:1,000 goat anti-mouse HRP (no. A28177; Invitrogen), 1 h at RT; 1:1,000 rabbit anti-CD178 (no. PA5-80617; Invitrogen), 1 h at RT; 1:1,000 rabbit anti-Cathepsin D (no. ab72915; Abcam), overnight at 4°C; and 1:5,000 anti-rabbit HRP (; no. 211-035-109; Jackson ImmunoResearch Laboratories Inc., West Grove, PA), 1 h at RT. After each primary antibody staining, the membrane was washed 3 × 5 min at RT with PBS containing 0.1% TWEEN-20 (PBST). After each secondary antibody staining, the membrane was washed for 5 × 5 min at RT. For each blot, the secondary HRP conjugates were detected using ProSignal Pico ECL Reagent (no. 20-300B; Genesee Scientific, San Diego, CA) and blot images were acquired with a FluorChem imager (ProteinSimple, San Jose, CA). After each HRP conjugate probing, the membrane was stripped in a buffer of 200 mM glycine, 0.1% SDS,1% TWEEN-20 pH 2.2 for 3 × 5 min followed by a quick wash with PBS pH 7.4 and a 2 × 10-min PBS pH 7.4 wash and then reequilibration into PBST through 3 × 5 min washes prior to reinitiation of blocking steps.

### Flow cytometry.

For the flow cytometry experiments, P2 cells (approximately 1 × 10^6^ cells per sample) were infected with pSV-CTFL-ΔΔ-S15-GFP-3′LTR virus and treated as described above for a pulse-chase assay with acid-stripping, except that anti-Env Fab probes BG18-Atto565 and PGT145-Atto565 in addition to b12-Atto565 were also included in the staining solution (each at 20 nM) with the aim of increasing signal for Env. After fixation and quenching, the cells were resuspended in DPBS and stored overnight in the dark at 4°C. Flow cytometry was performed using a BD LSR Fortessa instrument (BD Biosciences, San Jose, CA). GFP fluorescence was used to distinguish infected cells from uninfected cells in the infected sample. The intensities of Atto565 and AF647 fluorescence in each population of cells were analyzed using FlowJo software (BD Biosciences).

### Quantitative analysis of colocalization.

Quantitation of colocalization in pulse-chase assay images used the method of Fractional Overlap, or Manders’ Colocalization Coefficients (MCCs) (105). This method measures co-occurrence, essentially the extent to which pixels that are positive for one probe are also positive for the other probe, and was chosen for this application because cargo of a given compartment is expected to co-occur with a marker of that compartment but not necessarily codistribute with it (106). This method was selected rather than codistribution or extent to which the intensities of the two probes are proportional to each other, as measured by Pearson’s Correlation Coefficient. The image analysis was performed using custom code written in Matlab (Mathworks, Natick, MA). The steps of this analysis were as follows: (i) Local median background was subtracted from each image slice, using a 25 × 25 pixel neighborhood around each pixel for the local median calculation. (ii) The image stack was inspected in order to manually select a z-slice that was approximately equatorial for the individual cell to be analyzed. A range of 5 z-slices centered on the chosen slice was used for each cell. An regions of interest (ROI) defining the interior of the cell (excluding signal at or near the PM) was manually drawn on the chosen center slice. (iii) A residual background value, equal to the mean plus 1 standard deviation of the remaining nonzero intensities in the ROI, was subtracted from the ROI in each image slice. The background-subtracted ROI images from the 5 slices for each channel were concatenated next to each other, and binarized using the “*imbinarize”* function. The binarized ROI images were visually inspected, and if the signal in any of the channels was low enough that this process yielded mostly noise, then the ROI was excluded from analysis. (iv) MCCs were calculated between the binarized ROI images for each pair of channels. It should be noted that for any pair of channels *A* and *B*, there are two MCCs, *M1* and *M2*: *M1* = [pixels positive for both *A* and *B*]/[pixels positive for *A*], and *M2* = [pixels positive for both *A* and *B*]/[pixels positive for *B*] For our measurements of colocalization of Env with the various probes, the MCC with Env positive pixels in the denominator was used, so that the result does not depend on whether the other probe in question also occurs in other intracellular locations besides those where Env occurs. Statistical significance was assessed by Brown-Forsythe and Welch ANOVA tests and Dunnett’s T3 multiple-comparison test, using GraphPad Prism (GraphPad Software, La Jolla, CA).

### THP-1 macrophage and CEM-A T-cell virological synapse coculture system.

The THP-1 monocyte cell line was differentiated for 4 days using 100 ng·mL^−1^ phorbol 12-myristate 13-acetate (P1585; Sigma) in complete media on 25-mm glass coverslips in 3.5-cm culture dishes. On day 2, differentiating THP-1 macrophage were transduced with pSV-ΔΔΔ-CANTDeGFP-3′LTR (as described above), typically expressing HIV-1 proteins for 48 h prior to sample preparation. On day 5, undifferentiated THP-1 monocyctes were washed THP-1 macrophage were pulse-labeled for Env under conditions identical to those used for CEM-A. Briefly, THP-1 macrophage were pulse-labeled with anti-Env BG18 fab conjugated to the AF647 fluorescent dye (Thermo-Fisher) for 20 min under physiological culture conditions.

A CEM-A T-cell line stably expressing the membrane anchoring s15 peptide fused to TagRFP (CEM-A-Tag) was used for THP-1 macrophage coculture. For both acid-stripped and untreated conditions, CEM-A cells were added to dishes harboring macrophage on 25-mm coverslips (post-pulse-chase staining) and allowed to form synapses for 0.5–2.5 hours in the incubator. Virological synapses were imaging using spinning-disk confocal microscopy under the conditions described for CEM-A cells above. Virological synapse was here defined as a localization of Gag and Env from the infected cell within diffraction-limited proximity to the cell surface of the noninfected cell (marked with plasma membrane s15 marker).

For experiments using HIV-1-infected CEM-A cells and naive THP-1 macrophage, CEM-A cells were infected and pulse-labeled, and THP-1 monocytes were differentiated as described above. Briefly, 46–48 h after CEM-A infection cells were pulse-labeled with anti-Env BG18 Fab for 20 min, acid stripped to remove surface labeled-Env, dissociated from tissue culture plates using Cellstripper, washed with fresh RPMI media, and plated onto coverslips containing differentiated THP-1 macrophage (4 days postdifferentiation).

### Data availability.

All image analysis software and data will be made available upon request.
