# Supplementary material for: Endocytosed HIV-1 Envelope Glycoprotein Traffics to Rab14+ Late Endosomes and Lysosomes to Regulate Surface Levels in T-Cell Lines
Source: J Virol. 2022 Jun 30;96(14):e00767-22. doi: 10.1128/jvi.00767-22 (PMC9327703; doi:10.1128/jvi.00767-22)
Supplement: Supplemental file 1 — Fig. S1-S5. Download jvi.00767-22-s0001.pdf, PDF file, 1.1 MB [file jvi.00767-22-s0001.pdf]

Supplemental Data for

"Endocytosed HIV-1 envelope glycoprotein traffics to Rab14^{+} late endosomes and lysosomes to regulate surface levels in T-cell lines"

Huxley K. Hoffman[1,2], Rebekah S. Aguilar[1], Austin R. Clark[1], Nicholas S. Groves[1], Nairi Pezeshkian[1], Merissa M. Bruns[1], and Schuyler B. van Engelenburg[1*]

[1] Molecular and Cellular Biophysics Program, Department of Biological Sciences, University of Denver, Denver, CO 80210, USA

[2] Present address: Department of Cell and Developmental Biology, University of Colorado, Anschutz Medical Campus, Aurora, CO 80045, USA

*Author for correspondence (schuyler.vanengelenburg@du.edu)

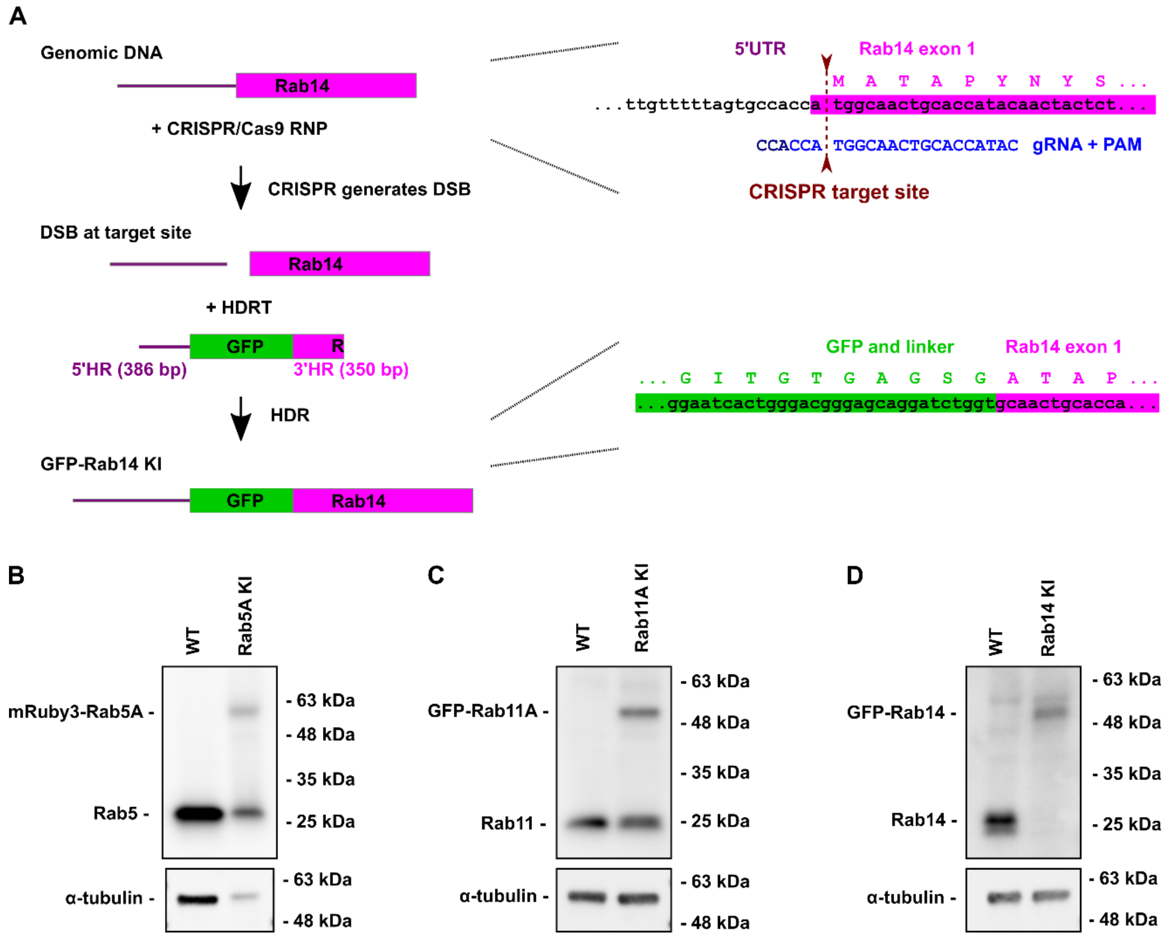

Supplemental Fig. S1. CRISPR/Cas9-mediated tagging of endogenous Rab5A, Rab11A, and Rab14 in T-cells. (A) FP tagging of endogenous Rabs via CRISPR/Cas9 and homology-directed repair (HDR). Rab14 is shown as an example; the strategies for Rab5A and Rab11A were similar. CEM-A T-cells were electroporated with CRISPR/Cas9 ribonucleoprotein (RNP) with guide RNA (gRNA) targeting the 5' end of the Rab gene, and a homology-directed repair (HDR) template containing the FP coding sequence flanked by homology regions (HR) for the target site. Cas9 generates a double-strand break (DSB) at the target site, allowing the template to be integrated at that site by HDR. This results in FP-Rab "knock-in" (KI), by which the FP-tagged Rab is expressed from its native genomic locus, avoiding overexpression. (B-D) Western blots for Rab5, Rab11, and Rab14 in the mRuby3-Rab5A KI, GFP-Rab11A KI, and GFP-Rab14 KI CEM-A cell lines respectively, showing the expression of the endogenously tagged proteins. Note that the expression of untagged Rab5 and Rab11 in the respective KI cell lines may be attributed to the other isoforms of these Rabs (Rab5B and Rab5C, and Rab11B) which were not targeted by the KIs.

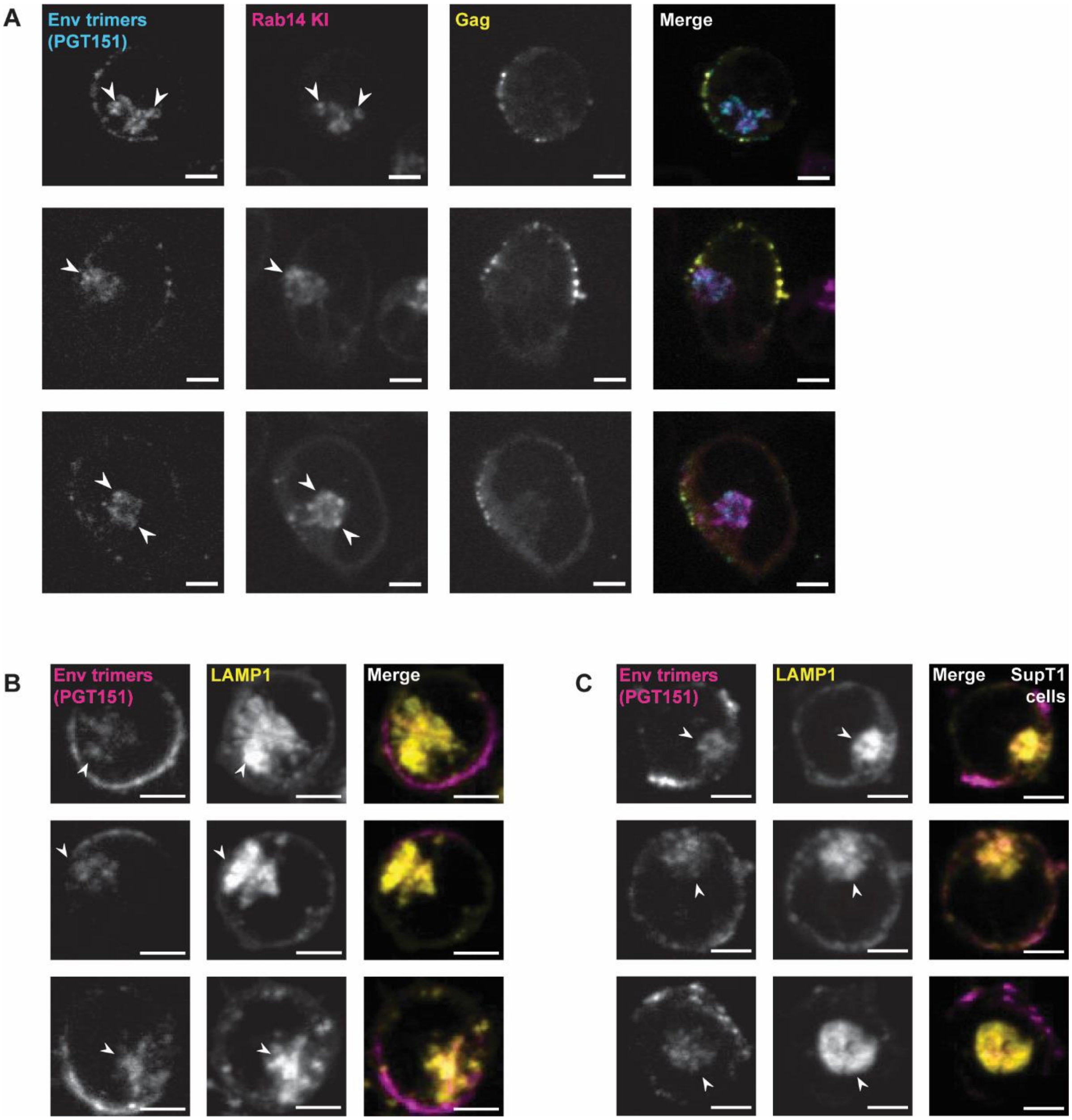

Supplemental Fig. S2. Mature trimers of HIV-1 Env traffic to Rab14$^+$ and LAMP1$^+$ compartments after endocytosis. (A) GFP-Rab14 KI CEM-A cells infected with HIV-1 virus pSV-ΔΔΔ-CANTD-TagRFP-3'LTR were pulse-labeled with anti-Env Fab PGT151-AF647 for 15 min, chased for 30 min, and imaged by live-cell confocal fluorescence microscopy. PGT151 recognizes a quaternary epitope of the heterodimeric gp120/gp41 cleaved (mature) trimer (46). PGT151-labeled Env (blue) colocalizes with endogenously tagged GFP-Rab14 (magenta; examples indicated with white arrows), confirming that functional trimers of HIV-1 Env traffic to Rab14$^+$ organelles upon endocytosis. As a marker of infection, HIV-1 Gag is labeled by the CANTD nanobody reporter (yellow). (B,C) WT CEM-A (B) or SupT1 (C) T-cells were infected with HIV-1 virus pSV-ΔΔΔ-LAMP1-Emerald-3'LTR, pulse-labeled with anti-Env Fab PGT151-AF647 as described above. In both CEM-A and SupT1 cells, internalized Env trimers localized to LAMP1$^+$ LEs/lysosomes. All scale bars 5 µm.

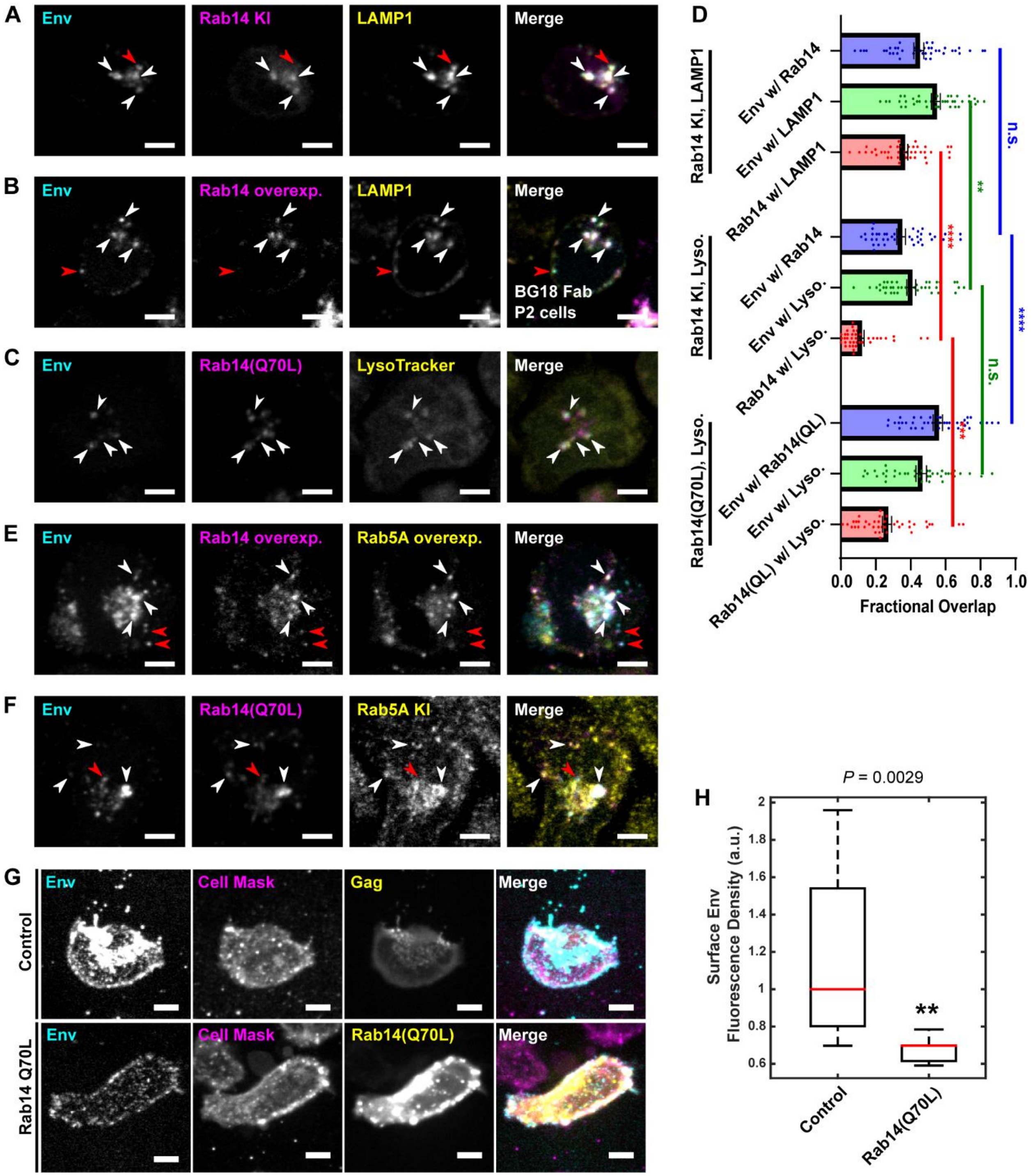

Supplemental Fig. S3. Active Rab14 colocalizes with internalized Env in early and late endosomal/lysosomal compartments and reduces Env surface expression.

(A-C,E-G) All scale bars 5 µm. White arrows indicate examples of colocalization, while red arrows indicate examples of non-colocalization. (A) GFP-Rab14 KI CEM-A T-cells were infected with HIV-1 virus pSV-ΔΔΔ-LAMP1-mRuby3-3'LTR. The cells were pulsed with anti-Env Fab b12-AF647 for 15 min at 4°C, chased at 37°C for 1 hour, fixed and imaged by confocal fluorescence microscopy. (B) P2 T-cells overexpressing low levels of TagRFP-Rab14 were infected with HIV-1 virus pSV-ΔΔΔ-LAMP1-Emerald-3'LTR, and pulse-labeled as in (A) except

using anti-Env Fab BG18-AF647. (C) WT CEM-A T-cells were infected with HIV-1 virus pSV-ΔΔΔ-Emerald-Rab14(Q70L)-3'LTR, which expresses a constitutively active Rab14 mutant. The cells were pulsed with anti-Env Fab b12-AF647 for 15 min at 4°C, chased at 37°C for 1 hour with LysoTracker Red DND-99 added in the media for the final 50 min, fixed, and imaged by confocal fluorescence microscopy. (D) Fractional overlap between probes in the assays represented in (A), Fig. 4A, and (C); n = 40 infected cells per sample. "Rab14 w/ LAMP1" for example denotes the percentage of Rab14-positive pixels that were also positive for LAMP1. Bars represent means, error bars represent s.e.m., and points represent the values for individual cells. Statistical significance was assessed by Brown-Forsythe and Welch ANOVA tests and Dunnett's T3 multiple comparisons test. n.s., $P > 0.05$; **, $P \leq 0.01$; ***, $P \leq 0.001$; ****, $P \leq 0.0001$.(E) CEM-A T-cells overexpressing low levels of TagRFP-Rab14 and GFP-Rab5A were infected with HIV-1 virus pSV-ΔΔΔ-3'LTRX, and pulse-labeled as in (A). (F) Rab5A KI CEM-A T-cells were infected with HIV-1 virus pSV-ΔΔΔ-Emerald-Rab14(Q70L)-3'LTR, and pulse-labeled as in (A). (G) CEM-A T-cells were infected with HIV-1 virus pSV-ΔΔΔ-CANTD-EGFP-3'LTR (control) or pSV-ΔΔΔ-Emerald-Rab14(Q70L)-3'LTR virus (Rab14(Q70L)). The cells were fixed but not permeabilized, and surface Env was stained using the full IgG anti-Env antibody b12-AF647, then imaged by confocal fluorescence microscopy. Representative single-slice images of the ventral surface of the cells are shown. (H) Quantification of cell-surface Env fluorescence density from the experiment shown in (G). N=21 cells for control and N=12 cells for Rab14(Q70L). Mean Env cellular fluorescence density is 1.11 ± 0.44 for control, and 0.68 ± 0.08 for Rab14(Q70L). Error represents std.

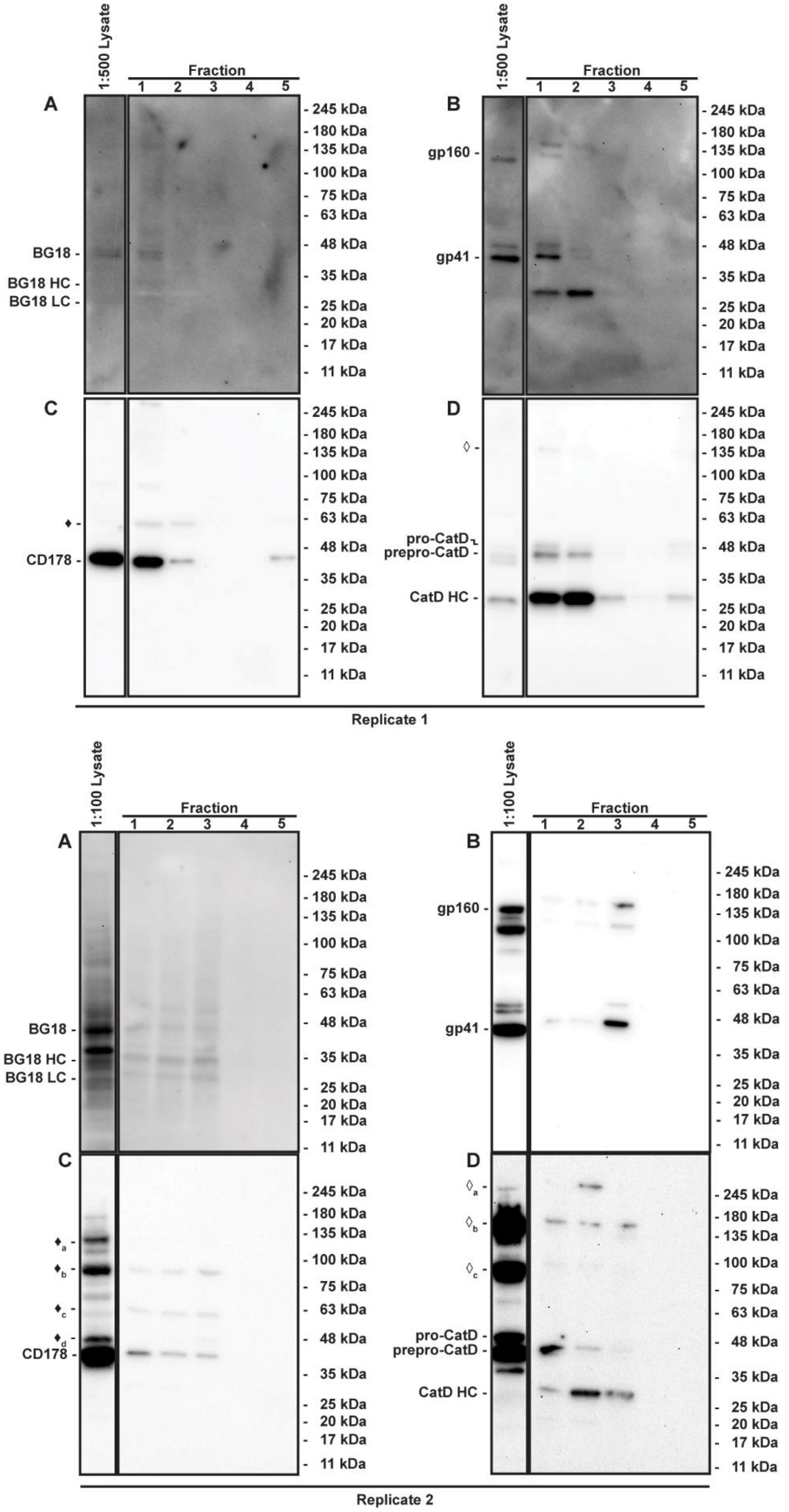

Supplemental Fig. S4. Biochemical detection of endocytosed Env in enriched lysosomal fractions. Uncropped western blots from the experiments shown in Fig. 5, taken from lysosome

isolation and enrichment of infected CEM-A cells pulse-chase labeled with the anti-Env Fab BG18. Lysosomes are enriched within the first two fractions (Replicate 1) or the first three fractions (Replicate 2). (A-B) Both anti-Env Fab BG18 bound to Env during pulse-chase, detected with anti-human λ light chain secondary antibody, and Env itself, detected by an anti-gp41 Env antibody (Chessie8), were detected within the lysosomal fractions. (C-D) Verification that fractions containing BG18 labeled Env are enriched for lysosome-like compartments, via probing with antibodies against endogenous lysosomal markers CD178 and cathepsin D (CatD). Uncharacterized bands labeled by the anti-CD178 and anti-Cathepsin D antibodies are labeled with a hollow (◊) or filled (♦) diamond. CD178, a type-II-membrane protein also known as FasL, is known to sort to secretory lysosomes and exists in either a membrane-associated (40-45 kDa) or soluble form (26 kDa). The band identified as ♦ (Rep. 1) or ♦$_c$ (Rep. 2) may be a complex formed between FasL and FasR. The band identified as ♦$_d$ (Rep. 2) may represent N-linked glycosylation of FasL. Identities of ♦$_a$ and ♦$_b$ are unknown. The inactive cathepsin D precuror protein, preprocathepsin D (prepro-CatD, 43 kDa), containing the secretion signal peptide is proteolytically processed upon entry into the secretory pathway, with subsequent glycosylation of the pro form, procathepsin D (pro-CatD, 46 kDa). After trafficking to lysosomes, pro-CatD is further cleaved to produce the mature, active cathepsin D heavy chain (HC, 28 kDa) and light chain (15 kDa). For the anti-Cathepsin D antibody, bands at ◊$_b$ and ◊$_c$ (150 and 90 kDa, respectively) are identified by the manufacturer as being uncharacterized and absent in a cathepsin knockout epithelial human cell line. The identity of ◊$_a$ is unknown. It is important to note that enrichment of each lysosomal marker, BG18 Fab, and Env gp41 was always observed in at least one of fractions 1-3 for five biological replicates performed on separate days. However, the relative levels of each species in a particular fraction (1-3) was found to be somewhat variable.

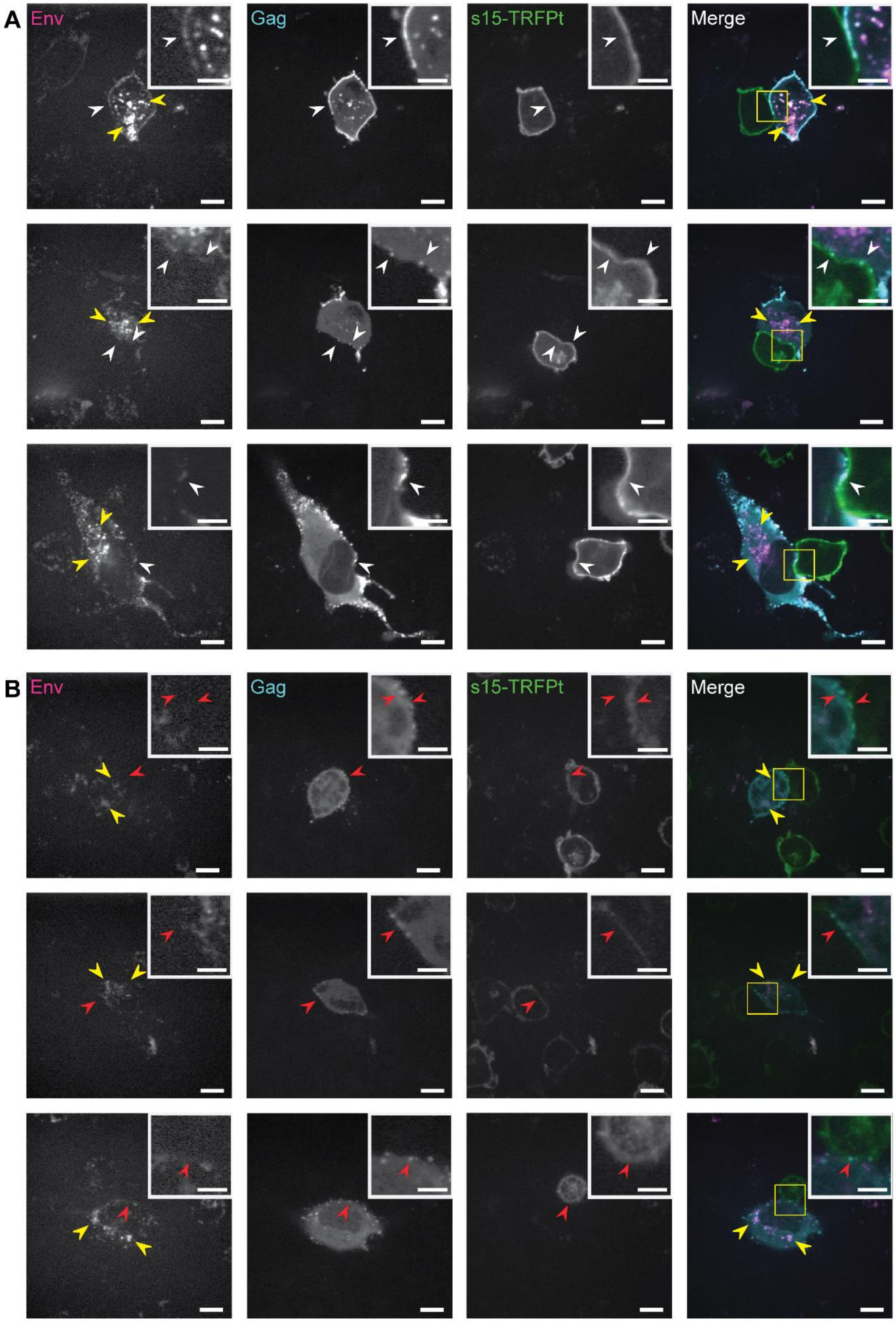

Supplemental Fig. S5. Virological synapses between infected THP-1 macrophages and naïve CEM-A T-cells display similar phenotypes when compared to reciprocal infection

experiments. (A) Representative examples of HIV-1 infected THP-1 macrophages forming virological synapses with naïve uninfected CEM-A T-cells. THP-1 macrophages were pulse-chase labelled with anti-Env BG18 Fab probe, acid treated to remove surface exposed Fab, and chased for 2-10 hours. While proximity was observed between THP-1 produced Gag (cyan) and our CEM-A T-cell membrane marker (green), there was very miniscule levels of BG18 fab (magenta) recruited to the synapse when compared to intracellular pulse-chased endocytic Env labelling. Endocytic Env residing proximal to virological synapses appeared to be docked in vesicular compartments just inside the macrophage membranes. (B) Non-acid stripped THP-1 macrophages displayed strong co-localization between T-cell membranes (green), Gag (cyan), and surface associated BG18 Fab (magenta). This suggests that surface labelled Env at the virological synapse, from infected THP-1 macrophage, is primarily associated with virus assembly sites. Similar to infected CEM-A T-cell virological synapses with naïve THP-1 macrophage, the majority of the endocytic pool of Env remains perinuclear, suggesting additional roles of Env endocytosis in promoting HIV-1 pathogenesis. White arrows indicate co-localization between Env, Gag, and CEM expressing PM associated s15-TagRFP, while red arrows indicate a lack of co-localization. Yellow arrows highlight the intracellular (endocytic) pool of Env that has not mobilized to the PM. Scale bars are 14 µm. Inset scale bars at 5 µm.
